# Physical activity across mid-life and mortality outcomes in Australian women: A target trial emulation using a prospective cohort

**DOI:** 10.1371/journal.pmed.1004976

**Published:** 2026-03-26

**Authors:** Binh Nguyen, Katherine B. Owen, Mengyun Luo, Wendy Brown, Gregore I. Mielke, Philip J. Clare, Ding Ding

**Affiliations:** 1 Prevention Research Collaboration, Sydney School of Public Health, Faculty of Medicine and Health, The University of Sydney, Camperdown, Australia; 2 Charles Perkins Centre, The University of Sydney, Camperdown, Australia; 3 School of Public Health, Faculty of Medicine, University of Queensland, Brisbane, Australia; 4 Faculty of Health Sciences and Medicine, Bond University, Gold Coast, Australia; 5 School of Human Movement and Nutrition Sciences, Faculty of Health and Behavioural Sciences, University of Queensland, Brisbane, Australia; 6 National Drug and Alcohol Research Centre, UNSW Sydney, Sydney, Australia; University of Leeds, UNITED KINGDOM OF GREAT BRITAIN AND NORTHERN IRELAND

## Abstract

**Background:**

Long-term causal evidence comparing different physical activity patterns and mortality outcomes is needed. Using observational data to emulate an RCT, this study compared different physical activity patterns over 15 years in relation to mortality from all causes, cardiovascular disease (CVD) and cancer in mid-aged Australian women.

**Methods and findings:**

A target trial emulation framework was used to emulate an RCT, based on data collected every 3 years (nine surveys between 1996 and 2019) from 11,169 women in the Australian Longitudinal Study on Women’s Health (ALSWH; 1946−51 cohort). Two emulated interventions were compared against consistent non-adherence (control) to WHO moderate-to-vigorous physical activity (MVPA) recommendations during the 15-year ‘exposure period’: (1) consistent adherence to recommendations (at least 150 min/week) over 15 years (2001−2016; women were 50−55–65−70 years); and (2) starting to meet the recommendations at age 55, 60, or 65 years. Analyses were adjusted for sociodemographic and health variables using marginal structural models with the assumptions of conditional exchangeability, positivity, consistency, and no interference. Mortality outcomes that occurred between surveys 4−9 (women were 53−58 to 68−73 years), were ascertained from Australian death registries. Comparing consistent adherence to MVPA recommendations with consistent non-adherence, there was evidence (Bayes factor [BF] = 5.71) for a protective effect for all-cause mortality (risk ratio [RR]: 0.50, 99.5% CI [0.27, 0.94]; risk difference [RD]: −5.2%, 99.5% CI [−10.5%, 0.1%]). Findings for CVD (BF = 2.05; RR: 0.50, 99.5% CI [0.19, 1.30]; RD: −2.1%, 99.5% CI [−5.3%, 1.1%]) and cancer mortality (BF = 2.26; RR: 0.35, 99.5% CI [0.10, 1.17]; RD: −3.3%, 99.5% CI [−8.4%, 1.9%]) were more uncertain and less conclusive, as were those for an effect of starting to meet MVPA recommendations in the mid-fifties on mortality outcomes. The main study limitations included reliance of self-reported physical activity and that findings may not be generalisable to all mid-aged Australian women.

**Conclusions:**

Based on findings from this target trial emulation, women should be encouraged to meet physical activity recommendations throughout mid-age to derive mortality benefits.

## Author summary

**Why was this study done?:**

- The evidence for an association between physical activity and mortality outcomes has generally been based on epidemiological studies relying on a single measurement of physical activity and not examining causal effects.
- Studies that examine the effect of longer-term patterns of physical activity on mortality outcomes are needed.

**What did the researchers do and find?:**

- Using a causal inference framework to imitate a randomised controlled trial based on observational data, this study offers long-term evidence comparing different physical activity patterns during mid-life in relation to all-cause, cardiovascular disease and cancer mortality in a cohort of Australian women.
- In this emulated target trial (*n* = 11,169), consistently meeting the World Health Organization’s recommendations of moderate-to-vigorous physical activity over 15 years was protective for all-cause mortality compared to consistent non-adherence to recommendations. Findings were uncertain and not conclusive for cardiovascular disease and cancer mortality, possibly due to insufficient statistical power from the smaller number of observed deaths from cardiovascular disease and cancer.
- Findings were also more uncertain and inconclusive for whether starting to meet guidelines earlier in mid-life (e.g., by the mid-fifties) resulted in lower risks of all-cause, cardiovascular disease and cancer mortality by the end of the study.

**What do these findings mean?:**

- Staying physically active at recommended levels throughout midlife provides protective benefits against premature mortality in women.
- This study supports the growing evidence that maintaining an active lifestyle in midlife provides health benefits.
- Limitations of the study included the use of self-reported physical activity, and findings may not apply to all mid-aged Australian women.

## Abstract 3

In a target trial emulation study, Binh Nguyen and colleagues use longitudinally collected data to investigate the impact of physical activity during mid-life on mortality of Australian women later in life.

## Introduction

Physical activity provides numerous health benefits and reduces the risk of chronic diseases and premature mortality [1–4]. The World Health Organization (WHO) recommends that adults undertake at least 150 min of weekly moderate-intensity physical activity and strength training involving major muscle groups on two or more days of the week [2]. Most epidemiological studies examining physical activity and subsequent health outcomes have relied on measuring physical activity at a single time point [5], failing to capture changing physical activity over time, and introducing measurement bias [5]. Physical activity levels change across the life span and can be influenced by major life transitions such as childbirth and retirement in women [6]. Studies that examine longer-term patterns of physical activity over several time points are needed to better understand how various patterns are related to health outcomes [7].

A few European and American studies have investigated the associations between trajectories of physical activity and mortality outcomes. These studies showed that physical activity participation in mid-life to older age was associated with lower risk of all-cause, cardiovascular disease (CVD) and cancer mortality irrespective of previous physical activity levels and other established risk factors [8–10]. However, these longitudinal studies might still be subject to biases due to factors such as complex confounding, and have not examined causal effects. Specifically, many confounders (e.g., adiposity) of the relationship between physical activity and mortality may themselves be affected by physical activity over time. For example, there is a bidirectional relationship between body mass index (BMI) and physical activity, and BMI could be both an outcome of early activity and exposure to later activity [11]. This type of bias cannot be sufficiently addressed using traditional analysis such as multivariable regression [12].

As conducting a physical activity randomised controlled trial (RCT) over many years is often not practical due to factors such as maintaining long-term compliance, and evidence from longer-term RCTs has been inconclusive [13,14], causal inference models have become increasingly recognised as an alternative for answering causal questions in health research [13,15]. Target trial emulation is a robust causal inference method that uses prospective cohort data to emulate an RCT to address some fundamental biases in observational studies [16–18]. By applying RCT principles to observational data, this approach can improve the clarity and relevance of both research questions and evidence [19,20]. This study is among the first to use a target trial framework to examine the effects of physical activity on mortality outcomes in the general population.

This study aimed to examine the effect of longitudinal patterns of physical activity over 15 years on mortality from all causes, CVD and cancer, in a population-based cohort of mid-aged Australian women. Using a target trial emulation framework, we tested the following hypotheses:

1. Consistently meeting WHO recommendations of moderate-to-vigorous physical activity (MVPA) [2] in all surveys of the exposure period (2001–2016, when women were between 50–55 and 65–70 years) (‘consistent adherence’) will result in lower risk of all-cause, CVD and cancer mortality than not meeting recommendations in any survey (control: ‘consistent non-adherence’).
2. Starting to meet the recommendations during the exposure period will result in lower mortality risk than the control condition of consistent non-adherence, and an earlier start in mid-age will have more favourable outcomes.

## Methods

### Target trial emulation

We used a target trial emulation approach to imitate a ‘target’ trial (i.e., the RCT that would have been conducted if this had been possible) from observational data [18]. The hypothetical ‘intervention’ was conceptualised as participants following different patterns of meeting recommendations for MVPA over the treatment/exposure period, compared with a ‘consistent non-adherence’ control (Table 1). The hypothetical intervention and control were not separate groups but rather the entire study sample observed under different exposures.

**Table 1:** Comparison between target randomised controlled trial (RCT) and trial emulated with Australian Longitudinal Study on Women’s Health (ALSWH) data.

	Target RCT	Emulated trial
Eligibility criteria	Women aged 47–52 years with unimpaired physical functioning and prepared to accept an intervention to meet MVPA recommendations	Women in the ALSWH cohort aged 47–52 years with unimpaired physical functioning who either did or did not meet MVPA recommendations
Trial arms	Intervention: Women adhering to meet MVPA recommendations over a 15-year period	Intervention^*^: Women meeting MVPA recommendations over a 15-year period (referred to hereafter as ‘exposed’)
	Control: Individuals caused to not meet MVPA recommendations	Control^*^: Individuals not meeting MVPA recommendations
Assignment procedures	Random assignment to either intervention or control arm at recruitment	Adjustment for confounding using targeted maximum likelihood estimation (TMLE)
Follow-up period	15 years of intervention period (6 triennial waves of follow-up), plus 3 years post-exposure for measurement of outcome	15 years of intervention period (6 triennial waves of follow-up), plus 3 years post-exposure for measurement of outcome
Outcomes	All-cause, cardiovascular disease-related and cancer-related mortality	All-cause, cardiovascular disease-related and cancer-related mortality
Causal effect measure	The difference in incidence risk in the intervention vs. the control study arms	Risk ratios and risk differences between the intervention and the control^*^
Statistical analysis	Estimate cumulative incidence of mortality over follow-up, using statistical analysis to adjust for baseline and post-baseline factors associated with adherence and loss to follow-up.	Statistical analysis same as per protocol in target trial with additional adjustment for pre-baseline factors associated with exposure.

* Note: The trial arms are not two separate groups but the entire study sample under different exposures.

Abbreviations: ALSWH, Australian Longitudinal Study on Women’s Health; MVPA, moderate-to-vigorous intensity physical activity; RCT, randomised controlled trial.

### Study population

**Fig 1:**
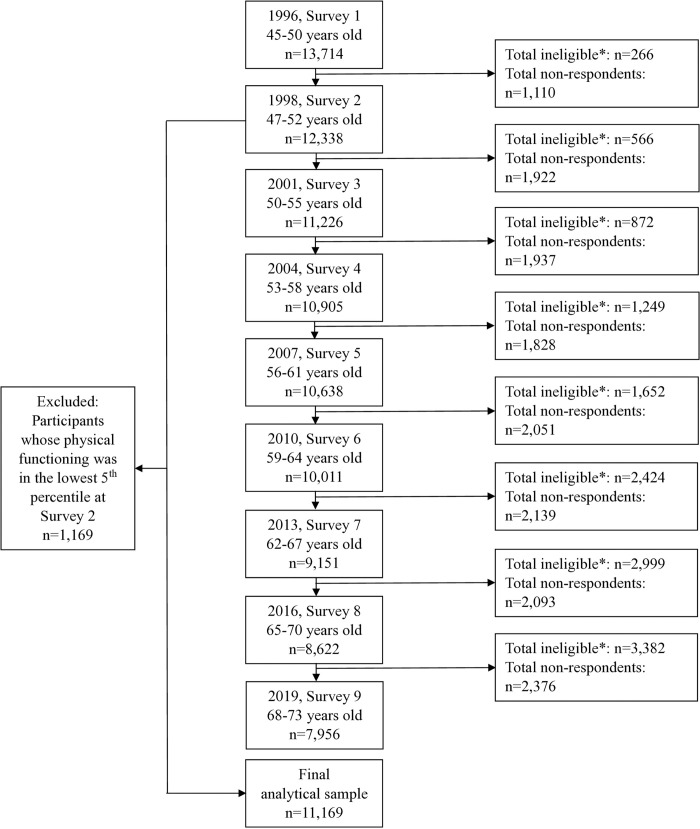
Study flow chart. *Ineligible includes: deceased, frailty and withdrawn.

The Australian Longitudinal Study on Women’s Health (ALSWH) is a population-based prospective cohort study, described in more detail on the website (www.alswh.org.au) and elsewhere [21,22]. The data for the present study were from nine surveys (1996−2019) conducted among the cohort born in 1946−1951. A national sample of women was randomly selected from Medicare Australia, the national insurance database, and mailed follow-up surveys approximately every three years thereafter (1998, 2001, 2004, 2007, 2010, 2013, 2016, 2019). The initial recruitment response rate was estimated to be 53%–56% [21]. Signed informed consent was obtained from all participants. We excluded participants with physical functioning in the lowest 5th percentile in 1998 (immediately prior to the exposure period) as their physical functioning may have been too compromised to participate in physical activity. Physical functioning was one of the subscales derived from the 36-item Medical Outcomes Study short-form survey (SF-36), which measures various aspects of health-related quality of life [23]. This study is reported as per the Transparent Reporting of Observational Studies Emulating a Target Trial (TARGET) guideline [24] (S1 TARGET Checklist), Strengthening the Reporting of Observational Studies in Epidemiology (STROBE) checklist (S1 STROBE Checklist), and the study flow chart is provided in Fig 1.

### Outcome variables

**Fig 2:**
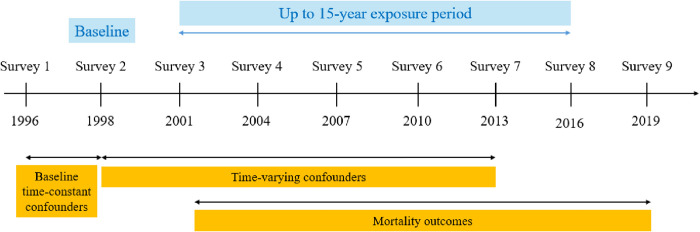
Study timeline.

The primary outcome is all-cause mortality, obtained from Australian death registries, with death obtained from the National Death Index (NDI) and cause of death from the Cause of Death (COD) database. Mortality was defined as a binary variable of whether death occurred in the period since completion of the previous survey (for example, a participant who completed survey 4 but subsequently died will be coded as having died at survey 5 and censored thereafter). CVD mortality was defined as death by CVD based on International Classification of Diseases 10th revision (ICD-10) codes I00-I99 as primary or secondary cause of death. Cancer mortality was limited to specific cancer types for which there was strong evidence, based on past research [25,26], including cancers in: bladder (C67), breast (C50), colorectal (C18-21), endometrium (C54.1), stomach (C16), kidney (C64), liver (C22), oesophagus (C15) and pancreas (C25). CVD and cancer mortality were similarly defined to all-cause mortality (as died at the survey subsequent to last completed survey), with participants who died from other causes censored at their time of death. Cause-specific mortality was estimated under the assumption that competing risks are independent. To address selection bias, censoring was handled using inverse probability of censoring weighting (IPCW). Mortality outcomes were considered between surveys 4–9 and expressed both in absolute (incidence risk [IR], risk differences [RDs]) and relative (risk ratio [RR]) terms. A timeline of the study is provided in Fig 2.

### Exposure measurement

Physical activity, measured using a reliable and validated modified version of the Active Australia survey questions [27,28], was calculated as the sum of reported minutes of walking (for exercise and transport), moderate leisure activity (e.g., social tennis, moderate exercise classes, recreational swimming, dancing), and vigorous leisure activity (e.g., aerobics, competitive sport, vigorous cycling, running, swimming; weighted double) per week [29]. Total physical activity was dichotomised as meeting or not meeting 150 min of weighted activity, based on WHO recommendations [2]. This investigation focussed solely on MVPA; data relating to strength training was not available. The exposure window corresponded to a 15-year period (2001–2016; surveys 3–8; age ranged from 50–55 to 65–70 years). Surveys 1 and 2 were not used due to different physical activity questions. Because measurement of the exposure was retrospective, we defined our time zero as wave 2, to ensure that exposure occurred *after* time zero and not before.

#### Emulated interventions.

We emulated two interventions, compared with consistent non-adherence to MVPA recommendations (control):

1 Consistent adherence to WHO recommendations of MVPA throughout the exposure period
2 Starting to meet the recommendations upon reaching 55, 60, or 65 years.

We evaluated the effect that would be expected if all participants followed these particular patterns of physical activity over the exposure period. We estimated the effect of meeting physical activity recommendations up to the end of the exposure period or the time of death, whichever happened first. Further details on the methods used can be found in S1 Text.

### Confounders

We explored probable causal relationships between confounders, exposures and outcomes using a Directed Acyclic Graph (DAG) (Fig A in S2 Text), to graphically present the relationships between variables [30], and to select confounders (variables identified as common causes of both exposures and outcomes). This included baseline (time-constant) confounders, measured in surveys 1 (1996) and 2 (1998), and included education and country of birth. Time-varying confounders were from surveys 2−7 (1998−2016), prior to each observation of the exposure. These included: age (continuous), employment status (employed; not employed), marital status (married/de facto; separated/divorced/never married), living with children (yes; no), area-level socioeconomic status, measured using the Index of Relative Socio-Economic Disadvantage (IRSD) (tertiles) [31], geographical remoteness based on the postcode-level Accessibility-Remoteness Index of Australia Plus (ARIA+) (major city; regional; remote) [32], lifetime risky alcohol consumption (>10 alcoholic drinks/week) based on the 2020 National Health Medical Research Council guidelines (yes; no) [33], heavy episodic alcohol consumption (>4 drinks on an occasion at least once a month) (yes; no), smoking status (never smoker; ex-smoker; current smoker), vegetable intake (<1; 1; 2; 3; 4; 5; ≥6 different vegetables/day), fruit intake (0; <1; 1; 2; 3; ≥4 pieces of fruit/day), Center for Epidemiological Studies-Depression (CES-D) scale (continuous) [34], perceived stress scale (continuous) [35], SF-36 subscale scores [23], BMI (underweight; healthy; overweight; obese), diagnosis/treatment history of coronary heart disease, stroke, arthritis, any cancer, anxiety, and depression (all yes; no). S2 Text presents detail of the confounders.

### Statistical analysis

We pre-registered our hypotheses and analysis plan (https://doi.org/10.17605/OSF.IO/PYTZX). We used the counterfactual framework, where each study participant has several *potential* outcomes (corresponding to different possible patterns of exposure), only one of which is *observed* (for the observed exposure history). Each possible pattern of exposure would be expected to lead to a specific outcome (death by the end of the study period, or not) for each participant—but only one such pattern is observed. More details about the counterfactual framework and its implementation are included in S1 Text.

Because the exposure (e.g., adherence to MVPA recommendations) in this study was not randomly allocated, emulating a target trial requires controlling for confounding [36]. In this case, some confounders may have been affected by past exposure (e.g., BMI_*t*−1_ is a confounder of MVPA at time *t*, but MVPA is also likely to affect subsequent BMI; as indicated in Fig A in S2 Text by the arrows connecting Exposure_*t*_ to Time − varying confounders_*t*_). To minimise bias due to exposure-affected time-varying confounding [11], we conducted all analyses using targeted maximum likelihood estimation (TMLE) [37]. TMLE is a consistent, doubly robust method for estimating causal effects in the presence of complex confounding, provided structural assumptions are met: conditional exchangeability [38], positivity [39], consistency [40], and no interference [41].

#### Assumptions for valid causal inference.

Marginal structural models, estimated via TMLE or otherwise, provide valid causal inference, under a set of structural assumptions. These are:

1. Conditional exchangeability: often called ‘no unmeasured confounding’, this assumption requires that exposure assignment is conditional only on measured confounders [38]. Based on literature in the field, we included the main sociodemographic and health confounders that are generally considered to be the primary common causes of physical activity and health outcomes [42,43]. Given that main confounders are included, and *E*-value analysis suggests that relatively substantial unmeasured confounding would be needed to alter the findings of the study, we believe that the conditional exchangeability assumption is satisfied. However, we acknowledge in the study limitations that the possibility remains that some confounders may have been missed.
2. Positivity: requires that all participants had at least some possibility of being exposed [39], although TMLE has been shown to be more robust to at least near violations of positivity [44]. This assumption is likely to hold. We excluded individuals with low physical function, and physical activity is a relatively common behaviour, so the chances that any subgroups in the data had a 0% or 100% chance of being exposed are very small.
3. Consistency: that there is no case where the observed outcome and the potential outcome under the observed exposure are different, which typically only occurs when the exposure is defined ambiguously [40]. Thus, this assumption is likely to hold in this case.
4. No interference: that the exposure of every participant is independent from the outcome of the other participants [41]. Interference typically happens when participants affect each other. As this is a population study, most participants will not meet or interfere with each other. Thus, the chances of participants interfering with each other are very low, and the validity of this assumption should not be an issue.

The longitudinal TMLE method used allowed us to estimate the effect of exposure at each time point, based on all observed information, and then use that information to estimate what we would expect to happen to each participant if they had followed a particular pattern of exposure, given their covariate history (for further detail see S1 Text).

Analyses were conducted on the Secure Unified Research Environment (SURE) platform in R 4.2.1, using the ‘ltmle’ package with models estimated via the SuperLearner, an ensemble machine learning algorithm [45]. All analyses were weighted based on the probability of selection into the study [16], to adjust for over-sampling of women in rural and remote areas. Results are reported as IRs, RRs, and RDs with 99.5% confidence intervals (CI), with the conservative alpha level (0.005) selected based on recommendations [46]. To assist in interpretation, we also estimated approximate Bayes factors (BFs), which indicate whether the observed results are more likely under the null or alternative hypothesis, taking into account the observed effect size, its standard error, and prior expectation about the hypothesis being tested. In this case, we have interpreted the BFs based on standard cut-points [47] and following the broad interpretation of Andraszewicz and colleagues [48]. Analysis code is available online (https://www.philipclare.com/code/alswh). S1 Text provides further details on the counterfactuals used, the estimation method, and BFs.

#### Secondary and sensitivity analyses.

We conducted a secondary analysis in which we examined the hypothetical interventions of ‘starting the exposure period meeting the MVPA recommendations but then stopping meeting them on reaching 55, 60, and 65 years’.

In addition, we conducted three sets of sensitivity analyses:

1. Using a lower cut-point of 75 min, to address the mostly curvilinear relationship between physical activity and health outcomes, where activity below the recommended levels providing significant health benefits [2,3].
2. Using the higher cut-point of 300 min of WHO MVPA recommendations.
3. *E*-value analysis (see S1 and S4 Text) to assess the potential impact of unmeasured confounding [49].

#### Missing data.

Missing data may occur due to (1) non-response to individual questions (intermittent missing data within surveys) and (2) non-response to any survey (including loss to follow-up). S3 Text describes the missing data and the procedures to address both forms of missing data, including multiple imputation [11] by chained equations (*M* = 40) [11], and data imputed using random forests [11]. All variables included in the analysis were included in the multiple imputation.

### Ethics approval

The ALSWH has ongoing ethical approval from the Human Research Ethics Committees of the Universities of Newcastle and Queensland (approval numbers H-076-0795 and 2004000224). Participants provided informed written consent to participate in the study.

## Results

### Sample characteristics

The analytical sample included 11,169 women, aged 45–50 years (mean [SD]: 49.5 [1.5]) in 1996 at survey 1. Unweighted and weighted sample characteristics at baseline are reported in Table 2. Most participants were born in Australia, employed, married, or in a de facto relationship. Nearly two thirds achieved less than a high school education, more than half lived with children (54.1%) and in regional areas, and almost a third resided in major cities. One in eight were lifetime risky drinkers, nearly one in six were current smokers, and almost half were overweight or obese.

**Table 2:** Characteristics of the study sample at baseline.

Variable	Categories	Mean (SD)/*n* (%)	
		(*N* = 11,169)	
		Unweighted	Weighted
**Age**		49.51 (1.46)	49.53 (1.82)
**Employment status**	Not employed	2,177 (19.5%)	2080 (19.0%)
	Employed	8,862 (79.3%)	8,878 (81.0%)
**Marital status**	Married/de facto	9,267 (83.0%)	8,983 (81.6%)
	Separated/divorced/never married	1,584 (14.2%)	1,769 (16.1%)
	Widowed	250 (2.2%)	255 (2.3%)
**Live with children under 18 years**	No	6,920 (62.0%)	6,598 (65.7%)
	Yes	3,313 (29.7%)	3,442 (34.3%)
**Live with children aged 18+ years**	No	6,676 (59.8%)	5,836 (57.8%)
	Yes	3,594 (32.2%)	4,260 (42.2%)
**Area-level SES (IRSD) in each SEIFA tertile**	Score for lowest tertile	941.41 (43.40)	938.79 (72.31)
	Score for middle tertile	985.97 (18.72)	985.27 (24.43)
	Score for highest tertile	1061.74 (73.00)	1071.58 (81.72)
**Remoteness (ARIA+)**	Major city	3,674 (32.9%)	7,181 (65.1%)
	Regional	6,882 (61.6%)	3,624 (32.8%)
	Remote	555 (5.0%)	230 (2.1%)
**Lifetime risky drinking** ^a^	No	8,829 (79.0%)	8,699 (86.3%)
	Yes	1,398 (12.5%)	1,380 (13.7%)
**Heavy episodic drinking** ^b^	No	7,017 (62.8%)	6,920 (66.9%)
	Yes	3,490 (31.2%)	3,421 (33.1%)
**Smoking status**	Never smoker	5,988 (53.6%)	5,914 (57.1%)
	Ex smoker	2,840 (25.4%)	2,797 (27.0%)
	Current smoker	1,694 (15.2%)	1,641 (15.9%)
**CESD-10 Depression Score**		6.13 (5.38)	6.20 (7.08)
**Stress Score (SD)**		0.60 (0.49)	0.62 (0.65)
**BMI Category**	Underweight	130 (1.2%)	134 (1.4%)
	Healthy	4,730 (42.3%)	4,832 (49.7%)
	Overweight	3,103 (27.8%)	2,995 (30.8%)
	Obese	1908 (17.1%)	1,756 (18.1%)
**Ever diagnosed/treated for…**	Heart disease	211 (1.9%)	204 (1.8%)
	Stroke	78 (0.7%)	78 (0.7%)
	Cancer	407 (3.6%)	440 (4.0%)
	Depression	1817 (16.3%)	1808 (16.3%)
	Anxiety	1,456 (13.0%)	1,497 (13.5%)
**Country of birth**	Australia	8,522 (76.3%)	7,849 (71.7%)
	Other	2,530 (22.7%)	3,104 (28.3%)
**Highest level of education**	Less than high school	7,183 (64.3%)	6,774 (61.8%)
	Trade/apprentice/certificate/diploma	2,223 (19.9%)	2,241 (20.4%)
	University	1,671 (15.0%)	1955 (17.8%)

Note: Percentages calculated as percent of total sample; percentages do not add to 100% due to missing data within variables.

^a^Lifetime risky alcohol consumption defined as >10 alcoholic drinks/week [25].

^b^Heavy episodic alcohol consumption defined as >4 alcoholic drinks on an occasion at least once a month [25].

Abbreviations: ARIA+, Accessibility-Remoteness Index of Australia Plus; BMI, body mass index; CESD-10, 10-item Centre for Epidemiological Studies Depression Scale; IRSD, Index of Relative Socio-Economic Disadvantage; SD, standard deviation.

### Main analyses

**Fig 3:**
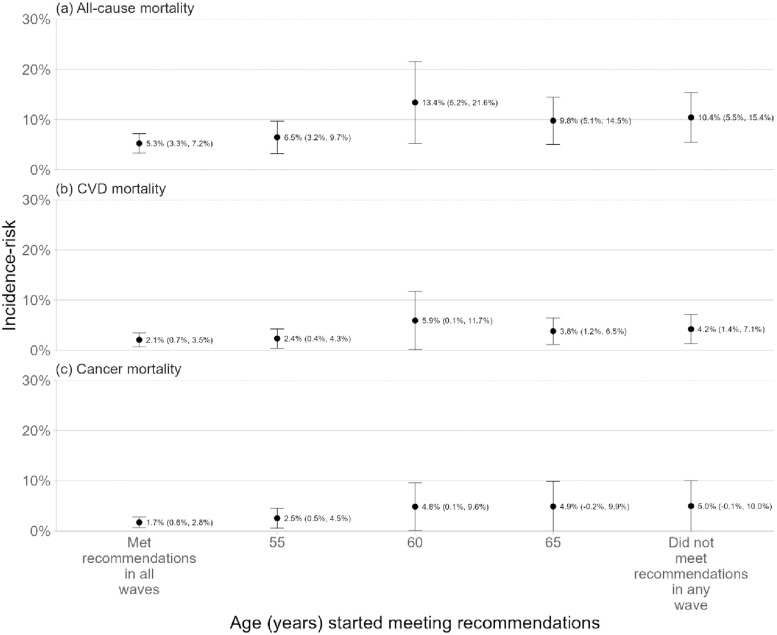
Incidence-risk of all-cause, cardiovascular disease (CVD), and cancer mortality linked to different ages of starting to meet moderate-to-vigorous intensity physical activity (MVPA) recommendations. Abbreviation: CVD, cardiovascular disease. The points represent the estimates and the bars the 99.5% confidence intervals. Numerical values have also been provided for the estimates and the confidence intervals (in parentheses). Models were adjusted for: highest level of education, country of birth, age, employment status, living with children, marital status, Socio-Economic Index For Areas Index of Relative Socio-Economic Disadvantage (SEIFA IRSD), geographical remoteness (Accessibility-Remoteness Index of Australia Plus, ARIA+), lifetime risky alcohol consumption, heavy episodic alcohol consumption, smoking status, vegetable intake, fruit intake, Center for Epidemiological Studies-Depression (CES-D) scale, perceived stress scale, SF-36 subscale scores, body mass index, diagnosis/treatment history of coronary heart disease, stroke, arthritis, any cancer, anxiety, and depression.

During the period when the outcomes were considered (surveys 4−9; aged between 53−58 and 68−73 years), 5.8% of the baseline sample died from all causes, 1.8% from CVD, and 1.7% from cancer (Table 3). The IR of all-cause, CVD and cancer mortality ranged respectively from 5.3% (99.5% CI [3.3%, 7.2%]), 2.1% (99.5% CI [0.7%, 3.5%]), and 1.7% (99.5% CI [0.6%, 2.8%]), when consistently meeting recommendations, to 10.4% (99.5% CI [5.5%, 15.4%]), 4.2% (99.5% CI [1.4%, 7.1%]) and 5.0% (99.5% CI [−0.1%, 10.0%]), when consistently not adhering to recommendations (Fig 3).

**Table 3:** Raw incidence of mortality between survey waves 4 (2004) and 9 (2019) when the outcomes were considered.

Number in sample/of deaths	Type of mortality	Survey wave number						Overall^a^
		4		5	6	7	8	9
*n* in sample^b^		10,295	9,880	9,391	8,740	8,157	7,319	10,295
Number (%) of deaths	All-cause	118 (1.1%)	108 (1.1%)	111 (1.2%)	125 (1.4%)	72 (0.9%)	110 (1.5%)	644 (6.3%)
	CVD	38 (0.4%)	26 (0.3%)	40 (0.4%)	34 (0.4%)	24 (0.3%)	36 (0.5%)	199 (1.9%)
	Cancer	31 (0.3%)	40 (0.4%)	31 (0.3%)	43 (0.5%)	21 (0.3%)	24 (0.3%)	189 (1.9%)

^a^Overall sample differs from ‘baseline’ sample due to participants who were censored prior to the exposure window.

^b^Participants were lost at each wave due to both death and loss to follow-up.

Abbreviation: CVD, cardiovascular disease.

RRs and RDs of all-cause, CVD and cancer mortality linked to different counterfactual patterns of MVPA are presented in Figs A and B in S4 Text. The risk of all-cause mortality associated with consistent adherence to MVPA recommendations was half that of consistent non-adherence (RR: 0.50, 99.5% CI [0.27, 0.94]; RD: −5.2%, 99.5% CI [−10.5%. 0.1%]). Based on the BF (Table A in S4 Text), there was evidence (BF = 5.71) for a protective effect of consistent adherence to MVPA recommendations on all-cause mortality. The effect size for consistent adherence to MVPA recommendations on the IR of CVD (BF = 2.05; RR: 0.50, 99.5% CI [0.19, 1.30]; RD: −2.1%, 99.5% CI [−5.3%, 1.1%]) and cancer mortality (BF = 2.26; RR: 0.35, 99.5% CI [0.10, 1.17]; RD: −3.3%, 99.5% CI [−8.4%, 1.9%]), compared to consistent non-adherence, was similar or stronger in magnitude to all-cause mortality. However, greater uncertainty made the findings less conclusive.

For other emulated patterns of physical activity that were considered, the effect sizes were of similar magnitude to those in the findings above. However, due to greater uncertainty, findings were also not conclusive for an effect of: starting to meet recommendations at age 55 on the IR of all-cause (BF = 2.16; RR: 0.62, 99.5% CI [0.31, 1.21]; RD: −4.0%, 99.5% CI [−9.7%, 1.8%]), CVD (BF = 1.56; RR: 0.56, 99.5% CI [0.20, 1.58]; RD: −1.9%, 99.5% CI [−5.2%, 1.5%]) and cancer (BF = 1.47; RR: 0.51, 99.5% CI [0.15, 1.79]; RD: −2.4%, 99.5% CI [−7.8%, 2.9%]) mortality; starting to meet recommendations at age 60 on the IR of all-cause (BF = 0.39; RR: 1.28, 99.5% CI [0.63, 2.57]; RD: 3.0, 99.5% CI [−5.9%, 11.8%]), CVD (BF = 0.59; RR: 1.38, 99.5% CI [0.46, 4.13]; RD: 1.7%, 99.5% CI [−4.3%, 7.8%]) and cancer (BF = 0.84; RR: 0.98, 99.5% CI [0.29, 3.26]; RD: −0.1%, 99.5% CI [−6.1%, 5.8%]) mortality. Although there was evidence for no effect of starting to meet recommendations at age 65 on the IR of all-cause mortality (BF = 0.31; RR: 0.94, 99.5% CI [0.80, 1.1]; RD: −0.7%, 99.5% CI [−2.3%, 1.0%]); the evidence was less clear for CVD (BF = 0.57; RR: 0.9%, 99.5% CI [0.63%, 1. 3%]; RD: −0.4%, 99.5% CI [−1.9%, 1.1%]) and cancer (BF = 0.34; RR: 0.98, 99.5% CI [0.76, 1.28]; RD: −0.1%, 99.5% CI [−1.6%, 1.4%]) mortality.

### Secondary analysis

Based on the BFs (Table A in S4 Text), findings were largely uncertain about the effect of starting the exposure period meeting the recommendations, but then stopping meeting them on reaching 55, 60, or 65 years on all outcomes, compared with control (Figs C–E in S4 Text).

### Sensitivity analysis

In sensitivity analyses using a lower threshold of 75 min/week, associations were similar to those of the main and secondary analyses (Figs F–K in S4 Text). For example, the RR for all-cause mortality comparing consistent adherence to recommendations with control was 0.40 (99.5% CI [0.20, 0.78]).

In sensitivity analyses using a higher threshold of 300 min/week, associations were also similar to those of the main analyses (Figs L–Q in S4 Text). For example, the RR for all-cause mortality comparing consistent adherence to recommendations with control was 0.65 (99.5% CI [0.35, 1.19]).

Findings from the main *E*-value analysis (Table B in S4 Text) showed that relatively strong unmeasured confounding (*E*-value = 3.38; i.e., any unmeasured confounder(s) would collectively need to more than triple the risk of both exposure and outcome) would be required to alter the findings of the effect of consistent adherence to MVPA recommendations on all-cause mortality. However, *E*-values for the exposures of starting to meet recommendations upon reaching 55, 60, or 65 were smaller for all mortality outcomes. Findings from the *E*-value analyses for secondary and sensitivity analyses are presented in Tables B–D in S4 Text.

## Discussion

Using a causal inference framework, this study found evidence that consistently meeting WHO MVPA recommendations throughout mid-age (approximately between 50 and 70 years, based on the exposure period in this study) was protective against premature mortality in women. Consistently meeting recommendations throughout mid-age was associated with half the IR of all-cause mortality compared with consistently not meeting the recommendations. Findings suggested a similar or stronger magnitude of effect in relation to CVD and cancer mortality, however due to greater uncertainty, findings were less conclusive for an effect of consistent adherence to MVPA recommendations on cause-specific mortality. In addition, there were inconclusive findings for whether starting to meet recommendations earlier in mid-life (e.g., early fifties) or stopping meeting recommendations later in mid-life (e.g., late sixties) were associated with lower risks of all-cause and cause-specific mortality.

Previous longitudinal studies have mostly examined physical activity trajectories and mortality outcomes [8,9]. In a large cohort study based on data from the National Institutes of Health-AARP Diet and Health Study, adults who increased leisure time physical activity in middle adulthood (40–61 years) had lower risks of all-cause (35%; 38% in women), CVD (43%; reported to be similar in women but results were not shown) and cancer mortality (16%; reported to be similar in women but results were not shown) [8]. Among 7,606 women aged 40–79 years from the European Prospective Investigation into Cancer and Nutrition-Norfolk cohort, followed for a median of 12.5 years, increasing physical activity trajectories were associated with a reduction of 27% for all-cause mortality, 31% for CVD mortality, and 11% for cancer mortality, compared to consistent inactivity irrespective of previous physical activity levels [9]. Among 3,231 men from the British Regional Heart study followed for a median of 16.4 years, compared with the low-decreasing physical activity trajectory group, being in the light-stable and moderate-increasing trajectory groups was associated with a lower risk of all-cause and CVD mortality [10]. The protective associations observed at lower physical activity levels in that study are in agreement with findings from our sensitivity analyses using a lower threshold of 75 min/week. The magnitude and direction of our findings align with those from previous studies. However, as our study used a robust causal inference framework to examine expected differences in mortality outcomes if all women followed a specific intervention during the exposure period, our findings are not directly comparable to those of previous studies which compared women following different patterns of physical activity.

The less conclusive findings for CVD and cancer mortality may be due to a lack of power resulting from the smaller number of CVD and cancer events. Alternatively, it is possible that previous studies overestimated the associations between physical activity and cause-specific mortality due to potential bias from statistical methods that did not adequately address complex confounding [13].

In addition to further strengthening the existing evidence for the health benefits of physical activity, our findings – derived using causal inference methods - provide evidence in women for the effects of consistently meeting MVPA recommendations in mid-age. Public health messages should emphasise the benefits for women of meeting physical activity recommendations throughout mid-age to derive longevity benefits. Our study found mostly inconclusive evidence relating to starting to meet or stopping meeting MVPA recommendations at various ages and mortality outcomes. The evidence from RCTs has been inconclusive as to the benefits of physical activity/exercise interventions initiated in older age on reducing the risk of premature mortality [13,50]. In addition, earlier analyses of ALSWH data using target trial emulation showed that earlier adoption of recommendations and maintaining meeting recommendations later in mid-life provided benefits for subsequent health-related quality of life among women [7]. The differences in findings may imply the effects of physical activity being outcome-specific or the current analysis being underpowered. Another likely reason is that the beneficial effects of starting to meet MVPA recommendations at, for example, 60 years of age, may become apparent over a longer duration beyond the follow-up period, but were not observed by the study end point of approximately 70 years (68–73 years).

Strengths of the study included adjusting for multiple, potential time-varying confounders, allowing the estimation of causal effects instead of associations, and applying causal inference framework with robust assumptions. We have taken extensive consideration to ensure the assumptions of causal inference can be best met. Although the weighted sample was broadly nationally representative at baseline, the sample may differ from the population in ways that cannot be easily adjusted via weighting to census data. In addition, due to the healthier sample of women remaining in the study [14], findings may not be generalisable to all mid-aged Australian women [51]. Physical activity was self-reported, although the survey instrument has shown acceptable validity and reliability [26,27]. The doubly robust method used was limited in the estimation of cause-specific mortality as it assumed that competing risks from other causes of death were independent. Another limitation of the study was the lack of available strength training data. Lastly, it is still possible that there was residual confounding, in which case the assumption of conditional exchangeability in the causal inference framework may not be met. However, the *E*-value analysis for the main analysis relating to all-cause mortality showed that substantial unmeasured confounding would be required to change the main findings.

Based on causal inference methods, this study demonstrates the protective effects of maintaining recommended levels of physical activity throughout mid-age on premature mortality. There were inconclusive findings on whether starting to meet recommendations by the mid-fifties resulted in lower mortality risk by the end of the study. Women should be encouraged to consistently engage in physical activity throughout the course of mid-age to derive longevity benefits.

## Supporting information

S1 TARGET Checklist Checklist downloaded from: https://target-guideline.org/. (DOCX)

S1 STROBE Checklist Strengthening the Reporting of Observational Studies in Epidemiology (STROBE) checklist (von Elm and colleagues, PLoS Medicine, 2007). The STROBE checklist is distributed under the Creative Commons Attribution License (CC BY 4.0): https://creativecommons.org/licenses/by/4.0/. Checklist is available at https://www.strobe-statement.org/.
(DOCX)

S1 Text Details of statistical methods. **Fig A in S1 Text.** Interpretation of Bayes factors using cut-points approximately equivalent to common frequentist critical *p*-values.
(DOCX)

S2 Text Confounder selection. **Fig A in S2 Text.** Directed acyclic graph showing the assumed causal structure.
(DOCX)

S3 Text Missing data. **Table A in S3 Text**. Summary of missing data in each analysis variable. **Fig A in S3 Text**. Most common patterns of missing data.
(DOCX)

S4 Text Additional results. **Fig A in S4 Text.** Risk ratio of all-cause, cardiovascular disease (CVD) and cancer mortality linked to different ages of starting to meet moderate-to-vigorous intensity physical activity (MVPA) recommendations versus not meeting recommendations at all. **Fig B in S4 Text.** Risk difference of all-cause, cardiovascular disease (CVD) and cancer mortality linked to different ages of starting to meet moderate-to-vigorous intensity physical activity (MVPA) recommendations versus not meeting recommendations at all. **Table A in S4 Text.** Estimation of Bayes factors. **Fig C in S4 Text.** Incidence-risk of all-cause, cardiovascular disease (CVD) and cancer mortality linked to different ages of stopping meeting moderate-to-vigorous intensity physical activity (MVPA) recommendations. **Fig D in S4 Text.** Risk ratio of all-cause, cardiovascular disease (CVD) and cancer mortality linked to different ages of stopping meeting moderate-to-vigorous intensity physical activity (MVPA) recommendations versus not meeting recommendations at all. **Fig E in S4 Text.** Risk difference of all-cause, cardiovascular disease (CVD) and cancer mortality linked to different ages of stopping meeting moderate-to-vigorous intensity physical activity (MVPA) recommendations versus not meeting recommendations at all. **Fig F in S4 Text.** Incidence-risk of all-cause, cardiovascular disease (CVD) and cancer mortality linked to different ages of starting to meet moderate-to-vigorous intensity physical activity (MVPA) recommendations—sensitivity analysis using 75 min/day. **Fig G in S4 Text.** Risk ratio of all-cause, cardiovascular disease (CVD) and cancer mortality linked to different ages of starting to meet moderate-to-vigorous intensity physical activity (MVPA) recommendations versus not meeting recommendations at all—sensitivity analysis using 75 min/day. **Fig H in S4 Text.** Risk difference of all-cause, cardiovascular disease (CVD) and cancer mortality linked to different ages of starting to meet moderate-to-vigorous intensity physical activity (MVPA) recommendations versus not meeting recommendations at all—sensitivity analysis using 75 min/day. **Fig I in S4 Text.** Incidence-risk of all-cause, cardiovascular disease (CVD) and cancer mortality linked to different ages of stopping meeting moderate-to-vigorous intensity physical activity (MVPA) recommendations—sensitivity analysis using 75 min/day. **Fig J in S4 Text.** Risk ratio of all-cause, cardiovascular disease (CVD) and cancer mortality linked to different ages of stopping meeting moderate-to-vigorous intensity physical activity (MVPA) recommendations versus not meeting recommendations at all – sensitivity analysis using 75 min/day. **Fig K in S4 Text.** Risk difference of all-cause, cardiovascular disease (CVD) and cancer mortality linked to different ages of stopping meeting moderate-to-vigorous intensity physical activity (MVPA) recommendations versus not meeting recommendations at all – sensitivity analysis using 75 min/day. **Fig L in S4 Text.** Incidence-risk of all-cause, cardiovascular disease (CVD) and cancer mortality linked to different ages of starting to meet moderate-to-vigorous intensity physical activity (MVPA) recommendations—sensitivity analysis using 300 min/day. **Fig M in S4 Text.** Risk ratio of all-cause, cardiovascular disease (CVD) and cancer mortality linked to different ages of starting to meet moderate-to-vigorous intensity physical activity (MVPA) recommendations versus not meeting recommendations at all—sensitivity analysis using 300 min/day. **Fig N in S4 Text.** Risk difference of all-cause, cardiovascular disease (CVD) and cancer mortality linked to different ages of starting to meet moderate-to-vigorous intensity physical activity (MVPA) recommendations versus not meeting recommendations at all—sensitivity analysis using 300 min/day. **Fig O in S4 Text.** Incidence-risk of all-cause, cardiovascular disease (CVD) and cancer mortality linked to different ages of stopping meeting moderate-to-vigorous intensity physical activity (MVPA) recommendations—sensitivity analysis using 300 min/day. **Fig P in S4 Text.** Risk ratio of all-cause, cardiovascular disease (CVD) and cancer mortality linked to different ages of stopping to meet moderate-to-vigorous intensity physical activity (MVPA) recommendations versus not meeting recommendations at all—sensitivity analysis using 300 min/day. **Fig Q in S4 Text.** Risk difference of all-cause, cardiovascular disease (CVD) and cancer mortality linked to different ages of stopping to meet moderate-to-vigorous intensity physical activity (MVPA) recommendations versus not meeting recommendations at all—sensitivity analysis using 300 min/day. **Table B in S4 Text.** E-value analysis for primary and secondary analyses. **Table C in S4 Text.** *E*-value analysis for sensitivity analysis using 75 min/day. **Table D in S4 Text.** *E*-value analysis for sensitivity analysis using 300 min/day.
(DOCX)

## Abbreviations

ALSWH: Australian Longitudinal Study on Women’s Health
ARIA+: Accessibility-Remoteness Index of Australia Plus
BF: Bayes factor
BMI: body mass index
CES-D: Center for Epidemiological Studies-Depression
CI: confidence interval
COD: Cause of Death
CVD: cardiovascular disease
DAG: Directed Acyclic Graph
ICD-10: International Classification of Diseases 10th revision
IPCW: inverse probability of censoring weighting
IR: incidence risk
IRSD: Index of Relative Socio-Economic Disadvantage
MVPA: moderate-to-vigorous physical activity
NDI: National Death Index
RCT: randomised controlled trial
RD: risk difference
RR: risk ratio
STROBE: Strengthening the Reporting of Observational Studies in Epidemiology
SURE: Secure Unified Research Environment
TARGET: Transparent Reporting of Observational Studies Emulating a Target Trial
TMLE: targeted maximum likelihood estimation
WHO: World Health Organization
