# Supplementary material for: Physical activity across mid-life and mortality outcomes in Australian women: A target trial emulation using a prospective cohort
Source: PLoS Med. 2026 Mar 26;23(3):e1004976. doi: 10.1371/journal.pmed.1004976 (PMC13020796; doi:10.1371/journal.pmed.1004976)
Supplement: S1 STROBE Checklist — The STROBE checklist is distributed under the Creative Commons Attribution License (CC BY 4.0): https://creativecommons.org/licenses/by/4.0/. Checklist is available at https://www.strobe-statement.org/. (DOCX) [file pmed.1004976.s002.docx]

# S1 STROBE Checklist

Strengthening the Reporting of Observational Studies in Epidemiology (STROBE) checklist.

| **Item** | **Item No.** | **Recommendation** | **Section** |  |  |  |
| --- | --- | --- | --- | --- | --- | --- |
| Title and abstract | 1 | (*a*) Indicate the study’s design with a commonly used term in the title or the abstract | Title page |  |  |  |
|  |  | (*b*) Provide in the abstract an informative and balanced summary of what was done and what was found | Abstract |  |  |  |
| Introduction | | | |  |  |  |
| Background/rationale | 2 | Explain the scientific background and rationale for the investigation being reported | Paragraphs 1 to 3 |  |  |  |
| Objectives | 3 | State specific objectives, including any prespecified hypotheses | Paragraph 4 |  |  |  |
| Methods | | | |  |  |  |
| Study design | 4 | Present key elements of study design early in the paper | Paragraph 1 |  |  |  |
| Setting | 5 | Describe the setting, locations, and relevant dates, including periods of recruitment, exposure, follow-up, and data collection | Study population |  |  |  |
| Participants | 6 | (*a*) Give the eligibility criteria, and the sources and methods of selection of participants. Describe methods of follow-up | Study population |  |  |  |
|  |  | (*b*) For matched studies, give matching criteria and number of exposed and unexposed |  |  |  |  |
| Variables | 7 | Clearly define all outcomes, exposures, predictors, potential confounders, and effect modifiers. Give diagnostic criteria, if applicable | Outcome variables, exposure measurement, confounders |  |  |  |
| Data sources/ measurement | 8* | For each variable of interest, give sources of data and details of methods of assessment (measurement). Describe comparability of assessment methods if there is more than one group | Same sections as above |  |  |  |
| Bias | 9 | Describe any efforts to address potential sources of bias | Statistical analysis |  |  |  |
| Study size | 10 | Explain how the study size was arrived at | Study population |  |  |  |
| Quantitative variables | 11 | Explain how quantitative variables were handled in the analyses. If applicable, describe which groupings were chosen and why | Outcome variables, exposure measurement, confounders, statistical analysis |  |  |  |
| Statistical methods | 12 | (*a*) Describe all statistical methods, including those used to control for confounding | Statistical analysis |  |  |  |
|  |  | (*b*) Describe any methods used to examine subgroups and interactions |  |  |  |  |
|  |  | (*c*) Explain how missing data were addressed | Statistical analysis |  |  |  |
|  |  | (*d*) If applicable, explain how loss to follow-up was addressed | Statistical analysis |  |  |  |
|  |  | (*e*) Describe any sensitivity analyses | Statistical analysis |  |  |  |
| Results | | | |  |  |  |
| Participants | 13* | (a) Report numbers of individuals at each stage of study—eg numbers potentially eligible, examined for eligibility, confirmed eligible, included in the study, completing follow-up, and analysed | Fig 1 |  |  |  |
|  |  | (b) Give reasons for non-participation at each stage | Fig 1 |  |  |  |
|  |  | (c) Consider use of a flow diagram | Fig 1 |  |  |  |
| Descriptive data | 14* | (a) Give characteristics of study participants (eg demographic, clinical, social) and information on exposures and potential confounders | Sample characteristics |  |  |  |
|  |  | (b) Indicate number of participants with missing data for each variable of interest | S3 Text |  |  |  |
|  |  | (c) Summarise follow-up time (eg, average and total amount) |  |  |  |  |
| Outcome data | 15* | Report numbers of outcome events or summary measures over time | Main analyses |  |  |  |
| Main results | 16 | (*a*) Give unadjusted estimates and, if applicable, confounder-adjusted estimates and their precision (eg, 95% confidence interval). Make clear which confounders were adjusted for and why they were included | Main analyses, sensitivity analyses, Fig 3, S4 Text |  |  |  |
|  |  | (*b*) Report category boundaries when continuous variables were categorized |  |  |  |  |
|  |  | (*c*) If relevant, consider translating estimates of relative risk into absolute risk for a meaningful time period |  |  |  |  |
| Other analyses | 17 | Report other analyses done—eg analyses of subgroups and interactions, and sensitivity analyses | Sensitivity analyses |  |  |  |
| Discussion | | | |  | | Discussion |
| Key results | 18 | Summarise key results with reference to study objectives | Paragraph 1 |  |  |  |
| Limitations | 19 | Discuss limitations of the study, taking into account sources of potential bias or imprecision. Discuss both direction and magnitude of any potential bias | Strengths and limitations |  |  |  |
| Interpretation | 20 | Give a cautious overall interpretation of results considering objectives, limitations, multiplicity of analyses, results from similar studies, and other relevant evidence | Discussion |  |  |  |
| Generalisability | 21 | Discuss the generalisability (external validity) of the study results | Strengths and limitations |  |  |  |
| Other information |  | | |  |  |  |
| Funding | 22 | Give the source of funding and the role of the funders for the present study and, if applicable, for the original study on which the present article is based | Funding statement |  |  |  |

Strengthening the Reporting of Observational Studies in Epidemiology (STROBE) checklist. (von Elm et al., PLoS Medicine, 2007). The STROBE checklist is distributed under the Creative Commons Attribution License (CC BY 4.0): https://creativecommons.org/licenses/by/4.0/. Checklist is available at https://www.strobe-statement.org/
