# Supplementary material for: Physical activity across mid-life and mortality outcomes in Australian women: A target trial emulation using a prospective cohort
Source: PLoS Med. 2026 Mar 26;23(3):e1004976. doi: 10.1371/journal.pmed.1004976 (PMC13020796; doi:10.1371/journal.pmed.1004976)
Supplement: S1 Text — Fig A in S1 Text. Interpretation of Bayes factors using cut-points approximately equivalent to common frequentist critical p-values. (DOCX) [file pmed.1004976.s003.docx]

# S1 Text: Details of statistical methods

## Counterfactuals

The estimands of this study are the expectations of the outcomes, under a set of counterfactual patterns of exposure, based on each treatment regime d, marginal with respect to observed confounders. Thus, each expected outcome can be thought of as what we would expect to happen if *all* participants in the study received that particular pattern of exposure, even if their actual exposure pattern was different (i.e. even if the pattern is ‘counter to fact’).

In this case, we have two sets of counterfactuals: two static counterfactual exposure patterns, and a set of dynamic exposure patterns.

The static counterfactual patterns considered are meeting MVPA recommendations in all six waves of the exposure period, versus meeting recommendations in none of the six waves:

$s=\{1,1,1,1,1,1\}$ (C-1)

for meeting recommendations in all waves, or

$s^{'}=\{0,0,0,0,0,0\}$ (C-2)

The dynamic treatment effects are based on age thresholds in the wave prior to each exposure, rather than being deterministic in each wave. For example:

$d\left( A_{t}=1 \right)=age_{t-1}>\theta$, (C-3)

where theta is the age threshold. Thus, it is possible for two participants to have slightly different patterns of exposure across waves of the study under dynamic regimes, if they reach the age threshold in different waves. This allows us to evaluate the effect of meeting MVPA recommendations upon reaching a particular age, but not prior to that, with a range of age thresholds considered (55, 60, and 65 years). Because of the age of the cohort during the study, the initial static exposure pattern can also be considered in this framework as age thresholds at the minimum age in wave 2 (44.6 years), and the maximum age in wave 7 (68.4 years).

## Targeted maximum likelihood estimation

Targeted maximum likelihood estimation (TMLE) is a method for estimating causal effects (1) that is ‘doubly robust’ because it uses two component models but requires only one of the two to be correctly specified.

TMLE starts by estimating an initial expectation $Q_{n}^{0}\left( A,L \right)$of the outcome using maximum likelihood (2, 3), similar to that created for the G-computation procedure (formula C-4), and a conditional expectation of the probability of exposure $g_{n}^{0}\left( A|L \right)$, equivalent to the propensity score model used by IPW (formula C-5).

$Q_{n}^{0}\left( A,L \right)=E\left( Y|A,L \right)$, (C-4)

$g_{n}^{0}\left( A|L \right)=logit\left( \frac{P\left( A|L \right)}{1-P\left( A|L \right)} \right)$, (C-5)

The propensity model is then used to create a ‘clever’ covariate $h$:

$h\left( A,L \right)=\frac{I\left( A=1 \right)}{g_{n}^{0}\left( 1 | L \right)}-\frac{I\left( A=0 \right)}{g_{n}^{0}\left( 0 | L \right)}$. (C-6)

The initial estimate of the outcome expectation $E\left( Y|A,L \right)$ is then ‘updated’ based on a function of the initial estimate and the clever covariate:

$Q_{n}^{*}\left( A,L \right)=Q_{n}^{0}\left( A,L \right)+\epsilon_{n}h\left( A,L \right)$, (C-7)

where $\epsilon_{n}$ is the coefficient of $h\left( A,L \right)$, and the coefficient of $Q_{n}^{0}\left( A,L \right)$ is constrained to be equal to one. The TMLE is then estimated by evaluating the final model at different treatment values,

$\theta_{n}^{TMLE}=\frac{1}{n}\sum_{i=1}^{n} Q_{n}^{*}\left( 1,L \right)-Q_{n}^{*}\left( 0,L \right)$. (C-8)

This estimator is equivalent to the G-computation estimator, except that the updated $Q_{n}^{*}\left( A,L \right)$ in place of the initial estimate $Q_{n}^{0}\left( A,L \right)$. In other words, TMLE is similar to the doubly robust method of using an IPTW outcome model to standardize the mean outcome via G-computation. This provides a targeted estimation of the target parameter (the effect of exposure), at the expense of bias in other parameters in the model (which are treated as nuisance parameters, and thus not reported).

## Longitudinal TMLE

While initially proposed for cross-sectional analyses, TMLE has been extended to longitudinal analysis, looking at the joint effect of repeated measurements of an exposure over time.(4)

It starts by sequentially estimating a series of models to estimate the effect of exposure on mortality at each time point in the exposure window (survey 3 to survey 8), controlling for confounders up to that wave and accounting for censoring via inverse probability of censoring weights.(5, 6) The final combined weights are based on the conditional probability of each participant receiving their observed exposure, and of being uncensored. That is, the weighted sample is *conditionally* balanced and uncensored. Starting at the final time-point t=T, it:

1. Estimates $E\left( Y_{t}|\bar{A}_{t-1},L_{t} \right)$ where $\bar{A}_{t}=(A_{0},\ldots,A_{t})$
2. Plugs in the counterfactual exposure based on the dynamic exposure rule
3. Estimate treatment and censoring models to construct a clever covariate $H\left( A,C,L \right)_{t-1}$ similar to formula C-6 in the single time case of TMLE, but extended to include the cumulative product of inverse exposure *and* censoring probabilities up to time t-1. and then update the predicted outcome using formula C-7.
4. Repeat steps 1-3, moving sequentially backward, and using the updated predicted value of $Y_{t}^{d}$ as the outcome for the next sequential iteration.

The final estimate of the causal effect is obtained from $Y_{t=1}^{d}$, and confidence intervals can be obtained from the influence curve. Further details of how longitudinal-TMLE models work and are fit can be found elsewhere.(6, 7)

All component models are fit using ensemble machine learning.(8)

In this study, we use this procedure to estimate mean expected probabilities of death under each of our static and dynamic counterfactual exposure patterns, such as those defined in formulas C-1 and C-2. We can then contrast intervention specific means under different regimes, $d$ and $d'$, to calculate additive or multiplicative effects equivalent to causal risk differences:

$RD=E\left( Y_{d} \right)-E\left( Y_{d^{'}} \right)$ (C-9)

or causal risk ratios:

$RR=E\left( Y_{d} \right)/E\left( Y_{d^{'}} \right)$. (C-10)

## Assumptions for Causal Inference

Marginal structural models, estimated via TMLE or otherwise, provide valid causal inference, under a set of structural assumption. These are:

1. Conditional exchangeability: often called ‘no unmeasured confounding’, this assumption requires that exposure assignment is conditional only on measured confounders.(9) Based on literature in the field, we included the main sociodemographic and health confounders that are generally considered to be the primary common causes of physical activity and health outcomes.(10, 11) Given that main confounders are included, and E-Value analysis suggests that relatively substantial unmeasured confounding would be needed to alter the significant findings of the study, we believe that the conditional exchangeability assumption is satisfied. However, we acknowledge in the study limitations that the possibility remains that some confounders may have been missed.
2. Positivity: requires that all participants had at least some possibility of being exposed (12), although TMLE has been shown to be more robust to at least near violations of positivity.(13) This assumption is likely to hold. We excluded individuals with low physical function, and physical activity is a relatively common behaviour, so the chances that any subgroups in the data had a 0% or 100% chance of being exposed are very small.
3. Consistency: that there is no case where the observed outcome and the potential outcome under the observed exposure are different, which typically only occurs when the exposure is defined ambiguously (14). Thus this assumption is likely to hold in this case.
4. No interference: that the exposure of every participant is independent from the outcome of the other participants (15). Interference typically happens when participants affect each other. As this is a population study, most participants will not meet or interfere with each other. Thus the chances of participants interfering with each other are very low, and the validity of this assumption should not be an issue.

## E-value analysis

E-value analysis is a sensitivity analysis developed to test the sensitivity of research findings to potential unmeasured confounding.(16)

Regarding continuous outcomes, the function uses the effect-size conversions proposed by Chinn (17) and VanderWeele (18) to approximately convert the mean difference between the exposed versus unexposed groups to the odds ratio that would arise from dichotomizing the continuous outcome.

For example, if resulting E-value is 2, this means that unmeasured confounder(s) would need to double the probability of a subject's being exposed versus not being exposed, and would also need to double the probability of being high versus low on the outcome, in which the cutoff for "high" versus "low" is arbitrary subject to some distributional assumptions.(17)

In this study, exposure is defined as a complex pattern of physical activity over time, and our effect of interest is the effect of that exposure on mortality in the study period. Thus, confounding can occur at multiple points – baseline confounders might effect PA and mortality across the whole study period, while time-varying confounders may affect only PA and mortality at later time-points. But both are able to affect the overall pattern of exposure. E-Value analysis makes no assumptions about the type or structure of confounding, only the magnitude required in order to alter the conclusions of the study.

## Bayes factors

Bayes factors are a metric that can be used to assess the strength of evidence for a statistical hypothesis. They are constructed as a ratio of the likelihoods of two ‘competing’ models (often under the null and the alternative hypotheses) and suggest whether one or the other is ‘more likely’ given the data. Unlike ‘p-values’, their construction means they are able to assess the strength of evidence for both the alternative and null hypotheses, where frequentists approaches can only assess evidence against the null, and thus cannot provide evidence about whether the null is likely to be ‘true’ (leading to the maxim ‘no evidence of effect is not the same as evidence of no effect’).

A Bayes factor of 1 suggests that the findings are equally likely under the null and alternative hypothesis. When framed as the ratio of evidence for the alternative hypothesis vs the null hypothesis, BFs <1 represent progressively more evidence for the null hypothesis, while BFs >1 represent progressively more evidence for the alternative hypothesis. While BFs are a continuous metric, they can be categorised with approximate equivalence to p-values.(19)

**Fig A in S1 Text** Interpretation of bayes factors using cut-points approximately equivalent to common frequentist critical p-values

In this study, our analyses were conducted under a frequentist approach, and approximate Bayes factors were estimating from the causal risk ratios produced by the model estimation, following the approach of Dienes,(20) and assuming a non-informative (uniform) prior distribution.

## References

1. van der Laan MJ, Rubin DB. Targeted maximum likelihood learning. The International Journal of Biostatistics. 2006;2(1). doi:10.2202/1557-4679.1043

2. Bembom O, Petersen ML, Rhee SY, Fessel WJ, Sinisi SE, Shafer RW, van der Laan MJ. Biomarker discovery using targeted maximum-likelihood estimation: application to the treatment of antiretroviral-resistant HIV infection. Statistics in Medicine. 2009;28(1). doi:10.1002/sim.3414

3. Arnold B, Arana B, Mausezahl D, Hubbard A, Colford JM. Evaluation of a pre-existing, 3-year household water treatment and handwashing intervention in rural Guatemala. International Journal of Epidemiology. 2009;38(6). doi:10.1093/ije/dyp241

4. van der Laan MJ, Gruber S. Targeted minimum loss based estimation of causal effects of multiple time point interventions. Int J Biostat. 2012;8(1). doi:10.1515/1557-4679.1370

5. Petersen M, Schwab J, Gruber S, Blaser N, Schomaker M, van der Laan M. Targeted Maximum Likelihood Estimation for Dynamic and Static Longitudinal Marginal Structural Working Models. J Causal Inference. 2014;2(2). doi:10.1515/jci-2013-0007

6. Schomaker M, Luque-Fernandez MA, Leroy V, Davies MA. Using longitudinal targeted maximum likelihood estimation in complex settings with dynamic interventions. Stat Med. 2019;38(24). doi:10.1002/sim.8340

7. Lendle SD, Schwab J, Petersen ML, van der Laan MJ. ltmle: An R Package Implementing Targeted Minimum Loss-Based Estimation for Longitudinal Data. Journal of Statistical Software. 2017;81(1). doi:10.18637/jss.v081.i01

8. Van der Laan MJ, Polley EC, Hubbard AE. Super learner. Statistical Applications in Genetics and Molecular Biology. 2007;6(1). doi:10.2202/1544-6115.1309

9. Greenland S, Pearl J, Robins JM. Causal diagrams for epidemiologic research. Epidemiology. 1999;10(1).

10. Lounassalo I, Salin K, Kankaanpää A, Hirvensalo M, Palomäki S, Tolvanen A, Yang X, Tammelin TH. Distinct trajectories of physical activity and related factors during the life course in the general population: a systematic review. BMC Public Health. 2019;19(1). doi:10.1186/s12889-019-6513-y

11. Daskalopoulou C, Stubbs B, Kralj C, Koukounari A, Prince M, Prina AM. Physical activity and healthy ageing: A systematic review and meta-analysis of longitudinal cohort studies. Ageing Res Rev. 2017;38. doi:10.1016/j.arr.2017.06.003

12. Petersen ML, Porter KE, Gruber S, Wang Y, van der Laan MJ. Diagnosing and responding to violations in the positivity assumption. Statistical Methods in Medical Research. 2012;21(1). doi:10.1177/0962280210386207

13. Porter KE. The Relative Performance of Targeted Maximum Likelihood Estimators Under Violations of the Positivity Assumption: UC Berkeley; 2011.

14. Cole SR, Frangakis CE. Commentary: the consistency statement in causal inference: a definition or an assumption? Epidemiology. 2009;20(1). doi:10.1097/EDE.0b013e31818ef366

15. Liu L, Hudgens MG, Becker-Dreps S. On inverse probability-weighted estimators in the presence of interference. Biometrika. 2016;103(4). doi:10.1093/biomet/asw047

16. VanderWeele TJ, Ding P. Sensitivity Analysis in Observational Research: Introducing the E-Value. Ann Intern Med. 2017;167(4). doi:10.7326/m16-2607

17. Chinn S. A simple method for converting an odds ratio to effect size for use in meta-analysis. Stat Med. 2000;19(22). doi:10.1002/1097-0258(20001130)19:22<3127::aid-sim784>3.0.co;2-m

18. VanderWeele TJ. On a Square-Root Transformation of the Odds Ratio for a Common Outcome. Epidemiology. 2017;28(6). doi:10.1097/ede.0000000000000733

19. Jeffreys H. The theory of probability: Oxford University Press; 1939.

20. Dienes Z. Using Bayes to get the most out of non-significant results. Front Psychol. 2014;5. doi:10.3389/fpsyg.2014.00781
