# Supplementary material for: Physical activity across mid-life and mortality outcomes in Australian women: A target trial emulation using a prospective cohort
Source: PLoS Med. 2026 Mar 26;23(3):e1004976. doi: 10.1371/journal.pmed.1004976 (PMC13020796; doi:10.1371/journal.pmed.1004976)
Supplement: S3 Text — Table A in S3 Text. Summary of missing data in each analysis variable. Fig A in S3 Text. Most common patterns of missing data. (DOCX) [file pmed.1004976.s005.docx]

# S3 Text: Missing data

There were a number of different mechanisms for missing data in the ALSWH dataset:

1. Loss to follow-up;
2. Questions not asked in some waves; and
3. Intermittent missingness due to refusal to answer, not knowing, etc.

The amount of missing data in each analysis variable is shown in Table E1. The most common patterns of missing data are shown in Figure E1. Data was confirmed to be not missing completely at random via Little’s test. Because of this, we assumed the data was missing at random. In this case, because outcomes were obtained through data linkage and were thus complete, all missingness was in the exposure and confounding variables. This means that complete case analysis is unlikely to introduce bias.(1) However, following the framework for the treatment of missing data proposed by the STRATOS initiative,(2) in order to increase efficiency and power, we conducted all analyses primary using multiple imputation.

Based on past research (3), we first attempted to impute using the ‘just another variable’ approach, in which data is imputed in ‘wide’ form, with one record per individual, and each repeated measurement of the same variable imputed as a separate variable. However, this approach failed to converge.

Because of this, we imputed the data using chained equations with the data in long form, using the R package ‘mice’ (4). To handle possible complexity in the data, we imputed all variables using random forests, from the package ‘ranger’ (5). Based on the proportion of missing information in the data, we used M=40 imputations (6). All variables used in the analysis models were included in imputation.

Analyses were then conducted on each imputed dataset, and combined using Rubin’s rules with the R package ‘Amelia’ (7).

As a sensitivity analysis, we also conducted analyses with variable imputed, but excluding those variables that were not asked in a specific wave. For example, questions about alcohol were not asked in wave 3 – in the primary analysis, this data is imputed, but in the sensitivity analysis, alcohol variables are included from wave 4 onwards, but not wave 3.

**Table A in S3 Text** Summary of missing data in each analysis variable.

|  |  | **Wave 1/2** | **Wave 3** | **Wave 4** | **Wave 5** | **Wave 6** | **Wave 7** | **Wave 8** |
| --- | --- | --- | --- | --- | --- | --- | --- | --- |
| Exposure | Physical activity | - | 1592 (14%) | 2121 (19%) | 2308 (21%) | 2821 (25%) | 3230 (29%) | 3702 (33%) |
| Time-varying confounders | Vegetable intake | 11169 (100%) | 11169 (100%) | 1540 (14%) | 1807 (16%) | 2293 (21%) | 11169 (100%) | - |
|  | Fruit intake | 11169 (100%) | 11169 (100%) | 1526 (14%) | 1795 (16%) | 2276 (20%) | 11169 (100%) | - |
|  | Alcohol - frequency | 942 (8%) | 11169 (100%) | 1822 (16%) | 2043 (18%) | 2517 (23%) | 3253 (29%) | - |
|  | Alcohol - binge drinking | 662 (6%) | 11169 (100%) | 1652 (15%) | 1819 (16%) | 2296 (21%) | 3038 (27%) | - |
|  | BMI | 1298 (12%) | 1794 (16%) | 2056 (18%) | 1990 (18%) | 2464 (22%) | 3218 (29%) | - |
|  | Employment status | 130 (1%) | 1786 (16%) | 1776 (16%) | 1960 (18%) | 2476 (22%) | 3066 (28%) | - |
|  | CES-D | 776 (7%) | 1557 (14%) | 1710 (15%) | 2140 (19%) | 2493 (22%) | 3030 (27%) | - |
|  | SEIFA | 65 (1%) | 1216 (11%) | 1628 (15%) | 1851 (17%) | 2327 (21%) | 2981 (27%) | - |
|  | Marital status | 68 (1%) | 1195 (11%) | 1641 (15%) | 1823 (16%) | 2308 (21%) | 3010 (27%) | - |
|  | Smoking status | 647 (6%) | 1199 (11%) | 1529 (14%) | 1791 (16%) | 2273 (20%) | 3001 (27%) | - |
|  | Live with children - under 18 | 936 (8%) | 1204 (11%) | 1530 (14%) | 1778 (16%) | 2280 (20%) | 3000 (27%) | - |
|  | Live with children - over 18 | 899 (8%) | 1200 (11%) | 1530 (14%) | 1777 (16%) | 2280 (20%) | 2998 (27%) | - |
|  | Mean stress | 650 (6%) | 1190 (11%) | 1557 (14%) | 1786 (16%) | 2281 (20%) | 2982 (27%) | - |
|  | ARIA+ | 58 (1%) | 1210 (11%) | 1514 (14%) | 1774 (16%) | 2264 (20%) | 2963 (27%) | - |
|  | Age | 0 (0%) | 1147 (10%) | 1503 (14%) | 1751 (16%) | 2248 (20%) | 2938 (26%) | - |
|  | 3-year diagnosis/treament for heart disease | 11169 (100%) | 1255 (11%) | 1767 (16%) | 1867 (17%) | 2342 (21%) | 2974 (27%) | - |
|  | 3-year diagnosis/treament for stroke | 11169 (100%) | 1255 (11%) | 1767 (16%) | 1867 (17%) | 2342 (21%) | 2974 (27%) | - |
|  | 3-year diagnosis/treament for cancer | 11169 (100%) | 1255 (11%) | 1767 (16%) | 1867 (17%) | 2342 (21%) | 3004 (27%) | - |
|  | 3-year diagnosis/treament for arthritis | 11169 (100%) | 1255 (11%) | 1767 (16%) | 1867 (17%) | 2342 (21%) | 2998 (27%) | - |
|  | 3-year diagnosis/treament for depression | 11169 (100%) | 1255 (11%) | 1767 (16%) | 1867 (17%) | 2342 (21%) | 2978 (27%) | - |
|  | 3-year diagnosis/treament for anxiety | 11169 (100%) | 1255 (11%) | 1767 (16%) | 1867 (17%) | 2342 (21%) | 2978 (27%) | - |
|  | SF-36^c^ | 178 (2%) | 1556 (14%) | 1870 (17%) | 2076 (19%) | 2383 (21%) | 3188 (29%) | - |
| Baseline/ time-constant confounders | Baseline - country of birth | 117 (1%) | - | - | - | - | - | - |
|  | Baseline - education | 92 (1%) | - | - | - | - | - | - |
|  | Baseline - ever diagnosis/treament for heart disease | 0 (0%) | - | - | - | - | - | - |
|  | Baseline - ever diagnosis/treament for stroke | 0 (0%) | - | - | - | - | - | - |
|  | Baseline - ever diagnosis/treament for cancer | 0 (0%) | - | - | - | - | - | - |
|  | Baseline - ever diagnosis/treament for arthritis | 0 (0%) | - | - | - | - | - | - |
|  | Baseline - ever diagnosis/treament for depression | 0 (0%) | - | - | - | - | - | - |
|  | Baseline - ever diagnosis/treament for anxiety | (0%) | - | - | - | - | - | - |

Abbreviations: ARIA+, Accessibility-Remoteness Index of Australia Plus; BMI, body mass index; CESD-10, 10-item Centre for Epidemiological Studies Depression Scale; IRSD, Index of Relative Socio-Economic Disadvantage; SF-36: 36-item Medical Outcomes Study short-form survey. Note: exposure was drawn from waves 3-8; time-varying confounders were drawn from waves 2-7; baseline/time-constant confounders were drawn from waves 1 and 2. ^a^ This was not asked in waves 3 and 7; ^b^ This was not asked in wave 3; ^c^ SF-36 is composed of 8 subscales, which were used for analysis, but missing data is reported for the measure overall because missing data was the same for each subscale.

**Fig A in S3 Text** Most common patterns of missing data.


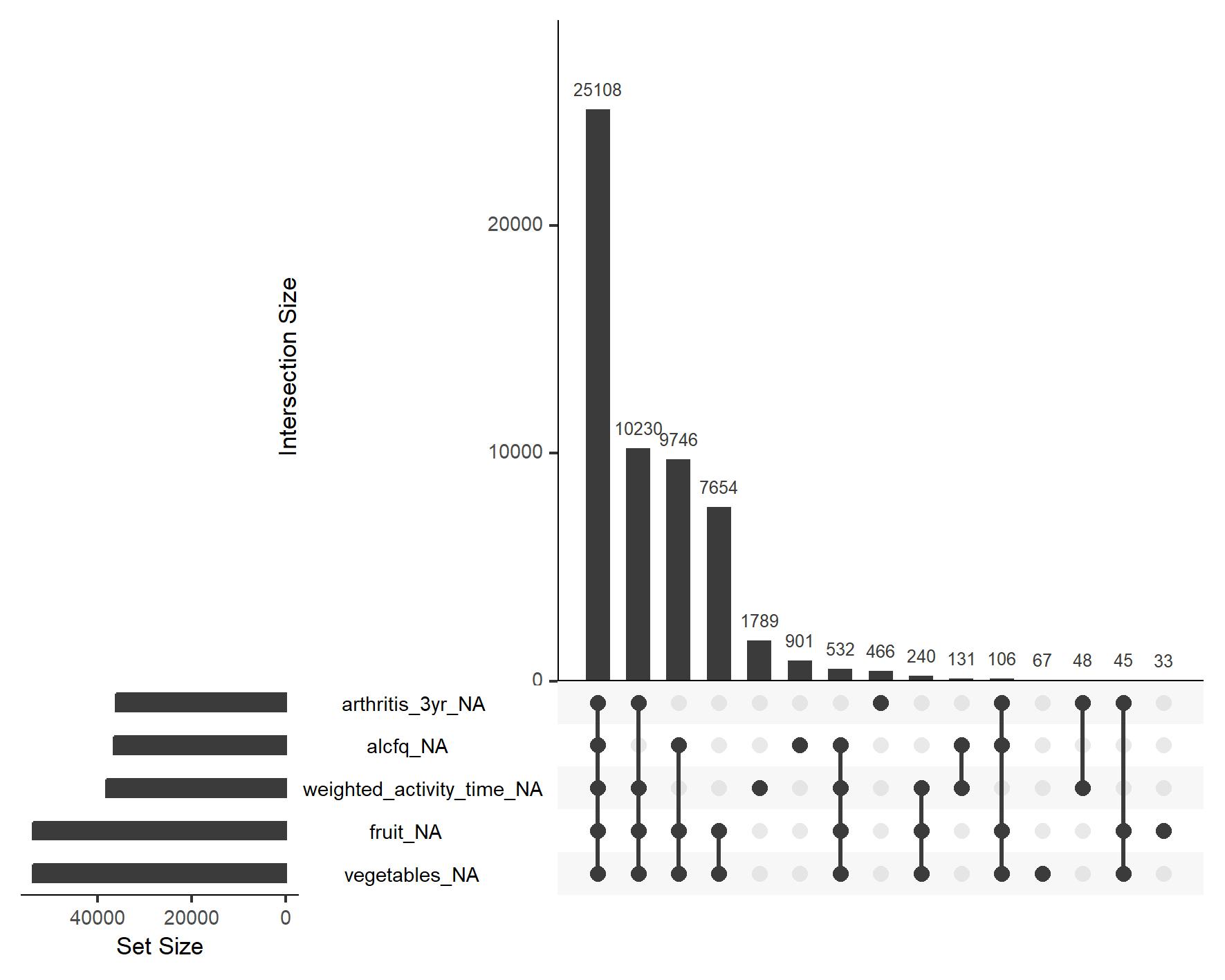


Abbreviations: alcfr, alcohol frequency.

Dots indicate the variable(s) that is/are missing. The lines joining the dots together represent the set of variables that are missing in the given pattern.

References

1. Hughes RA, Heron J, Sterne JAC, Tilling K. Accounting for missing data in statistical analyses: multiple imputation is not always the answer. International Journal of Epidemiology. 2019;48(4):1294-304. doi:10.1093/ije/dyz032.

2. Lee KJ, Tilling KM, Cornish RP, Little RJA, Bell ML, Goetghebeur E, et al. Framework for the treatment and reporting of missing data in observational studies: The Treatment And Reporting of Missing data in Observational Studies framework. Journal of Clinical Epidemiology. 2021;134:79-88. doi:10.1016/j.jclinepi.2021.01.008.

3. Huque MH, Carlin JB, Simpson JA, Lee KJ. A comparison of multiple imputation methods for missing data in longitudinal studies. BMC Medical Research Methodology. 2018;18(1):168. doi:10.1186/s12874-018-0615-6.

4. van Buuren S, Groothuis-Oudshoorn K. mice: multivariate imputation by chained equations in R. Journal of Statistical Software. 2011;45(3). doi:10.18637/jss.v045.i03.

5. Wright MN, Wager S, Probst P. Ranger: A fast implementation of random forests. R package version 012. 2020;1.

6. Graham JW, Olchowski AE, Gilreath TD. How many imputations are really needed? Some practical clarifications of multiple imputation theory. Prevention Science. 2007;8(3):206-13. doi:10.1007/s11121-007-0070-9.

7. Honaker J, King G, Blackwell M. Amelia II: a program for missing data. Journal of Statistical Software. 2011;45(7):47. doi:10.18637/jss.v045.i07.
