# Supplementary material for: Physical activity across mid-life and mortality outcomes in Australian women: A target trial emulation using a prospective cohort
Source: PLoS Med. 2026 Mar 26;23(3):e1004976. doi: 10.1371/journal.pmed.1004976 (PMC13020796; doi:10.1371/journal.pmed.1004976)
Supplement: S4 Text — Fig A in S4 Text. Risk ratio of all-cause, cardiovascular disease (CVD) and cancer mortality linked to different ages of starting to meet moderate-to-vigorous intensity physical activity (MVPA) recommendations versus not meeting recommendations at all. Fig B in S4 Text. Risk difference of all-cause, cardiovascular disease (CVD) and cancer mortality linked to different ages of starting to meet moderate-to-vigorous intensity physical activity (MVPA) recommendations versus not meeting recommendations at all. Table A in S4 Text. Estimation of Bayes factors. Fig C in S4 Text. Incidence-risk of all-cause, cardiovascular disease (CVD) and cancer mortality linked to different ages of stopping meeting moderate-to-vigorous intensity physical activity (MVPA) recommendations. Fig D in S4 Text. Risk ratio of all-cause, cardiovascular disease (CVD) and cancer mortality linked to different ages of stopping meeting moderate-to-vigorous intensity physical activity (MVPA) recommendations versus not meeting recommendations at all. Fig E in S4 Text. Risk difference of all-cause, cardiovascular disease (CVD) and cancer mortality linked to different ages of stopping meeting moderate-to-vigorous intensity physical activity (MVPA) recommendations versus not meeting recommendations at all. Fig F in S4 Text. Incidence-risk of all-cause, cardiovascular disease (CVD) and cancer mortality linked to different ages of starting to meet moderate-to-vigorous intensity physical activity (MVPA) recommendations—sensitivity analysis using 75 min/day. Fig G in S4 Text. Risk ratio of all-cause, cardiovascular disease (CVD) and cancer mortality linked to different ages of starting to meet moderate-to-vigorous intensity physical activity (MVPA) recommendations versus not meeting recommendations at all—sensitivity analysis using 75 min/day. Fig H in S4 Text. Risk difference of all-cause, cardiovascular disease (CVD) and cancer mortality linked to different ages of starting to meet moderate-to-vigorous intens [file pmed.1004976.s006.docx]

# S4 Text: Additional results

**Fig A in S4 Text** Risk ratio of all-cause, cardiovascular disease (CVD) and cancer mortality linked to different ages of starting to meet moderate-to-vigorous intensity physical activity (MVPA) recommendations versus not meeting recommendations at all.


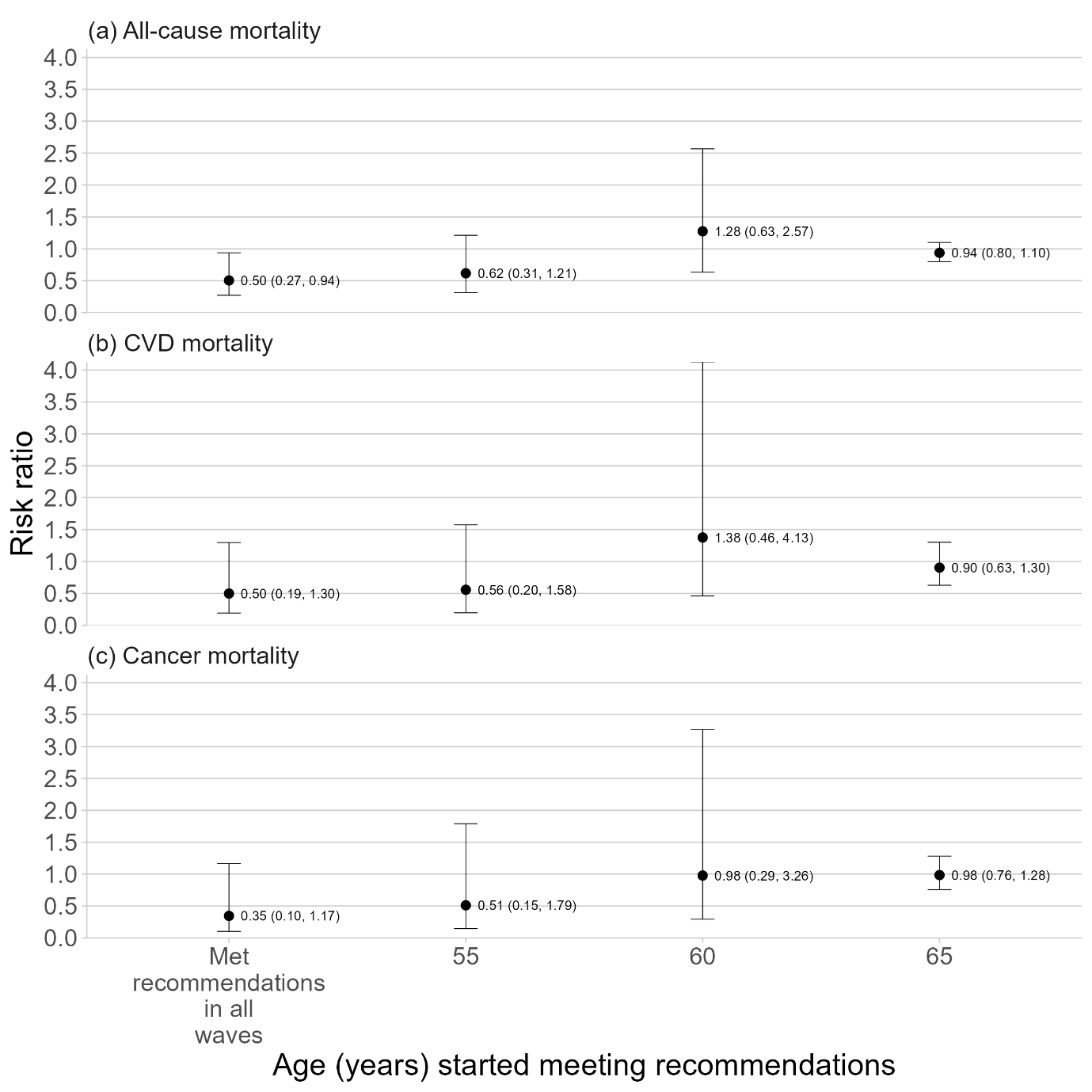


Abbreviations: CVD, cardiovascular disease.

The points represent the estimates and the bars the 99.5% confidence intervals. Numerical values have also been provided for the estimates and the confidence intervals (in parentheses).

Models were adjusted for: highest level of education, country of birth, age, employment status, living with children, marital status, Socio-Economic Index For Areas Index of Relative Socio-Economic Disadvantage (SEIFA IRSD), geographical remoteness (Accessibility-Remoteness Index of Australia Plus, ARIA+), lifetime risky alcohol consumption, heavy episodic alcohol consumption, smoking status, vegetable intake, fruit intake, Center for Epidemiological Studies-Depression (CES-D) scale, perceived stress scale, SF-36 subscale scores, body mass index, diagnosis/treatment history of coronary heart disease, stroke, arthritis, any cancer, anxiety, and depression.

**Fig B in S4 Text** Risk difference of all-cause, cardiovascular disease (CVD) and cancer mortality linked to different ages of starting to meet moderate-to-vigorous intensity physical activity (MVPA) recommendations versus not meeting recommendations at all.


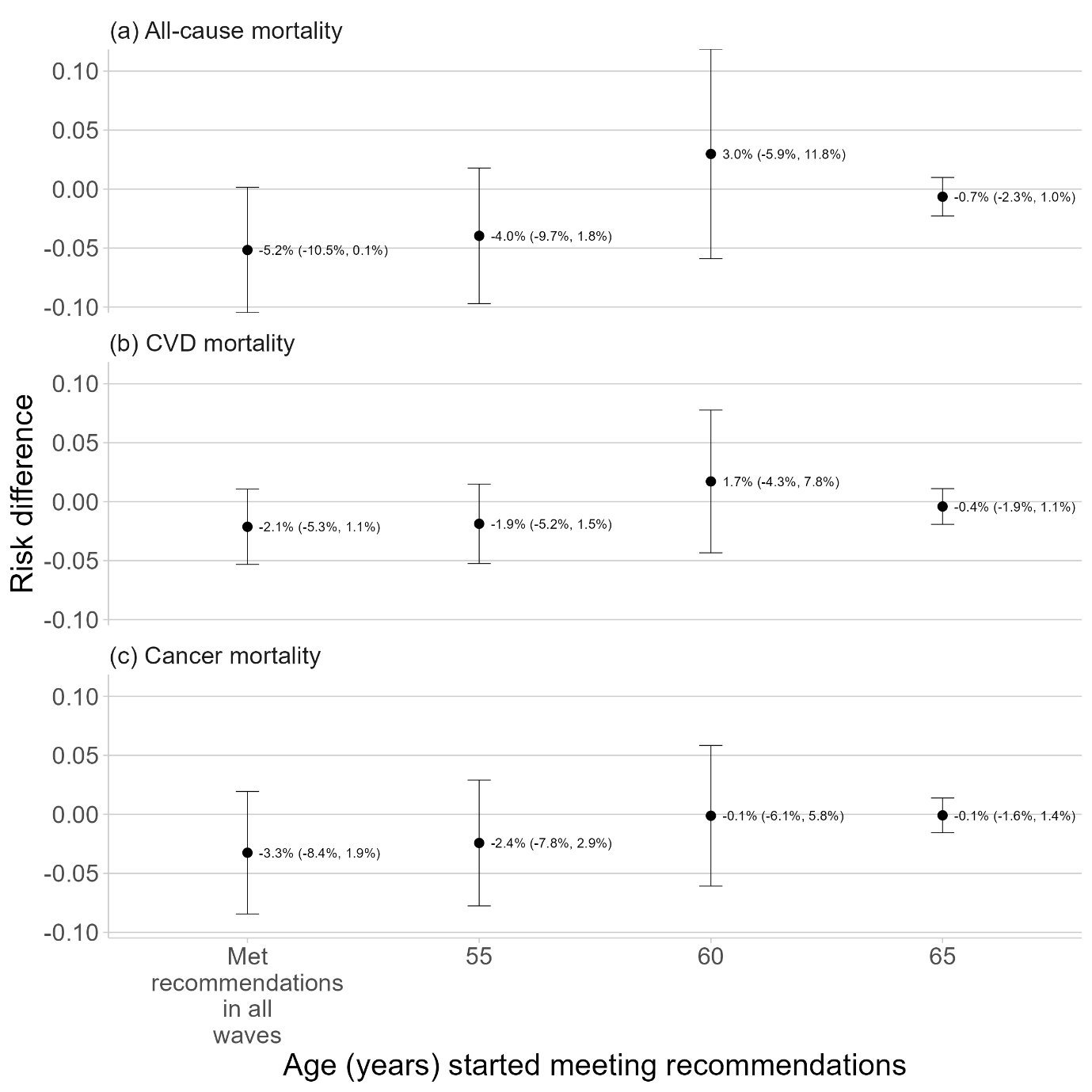


Abbreviations: CVD, cardiovascular disease.

The points represent the estimates and the bars the 99.5% confidence intervals. Numerical values have also been provided for the estimates and the confidence intervals (in parentheses).

Models were adjusted for: highest level of education, country of birth, age, employment status, living with children, marital status, Socio-Economic Index For Areas Index of Relative Socio-Economic Disadvantage (SEIFA IRSD), geographical remoteness (Accessibility-Remoteness Index of Australia Plus, ARIA+), lifetime risky alcohol consumption, heavy episodic alcohol consumption, smoking status, vegetable intake, fruit intake, Center for Epidemiological Studies-Depression (CES-D) scale, perceived stress scale, SF-36 subscale scores, body mass index, diagnosis/treatment history of coronary heart disease, stroke, arthritis, any cancer, anxiety, and depression.

**Table A in S4 Text** Estimation of Bayes factors.

| **Counterfactual** | | **All-cause mortality** | **CVD mortality** | **Cancer mortality** |
| --- | --- | --- | --- | --- |
|  |  | **At estimate** | **At estimate** | **At estimate** |
| Age started meeting recommendations | Met recommendations in all waves | 5.71 | 2.05 | 2.26 |
|  | 55 | 2.16 | 1.56 | 1.47 |
|  | 60 | 0.39 | 0.59 | 0.84 |
|  | 65 | 0.31 | 0.57 | 0.34 |
|  | Did not meet recommendations in any wave | REF | REF | REF |
| Age stopped meeting recommendations | Did not meet recommendations in any wave | REF | REF | REF |
|  | 55 | 1.23 | 0.72 | 1.58 |
|  | 60 | 2.11 | 2.10 | 1.26 |
|  | 65 | 1.82 | 1.76 | 1.83 |
|  | Met recommendations in all waves | 5.71 | 2.05 | 2.26 |

Abbreviations: CVD, cardiovascular disease.

**Fig C in S4 Text** Incidence-risk of all-cause, cardiovascular disease (CVD) and cancer mortality linked to different ages of stopping meeting moderate-to-vigorous intensity physical activity (MVPA) recommendations.


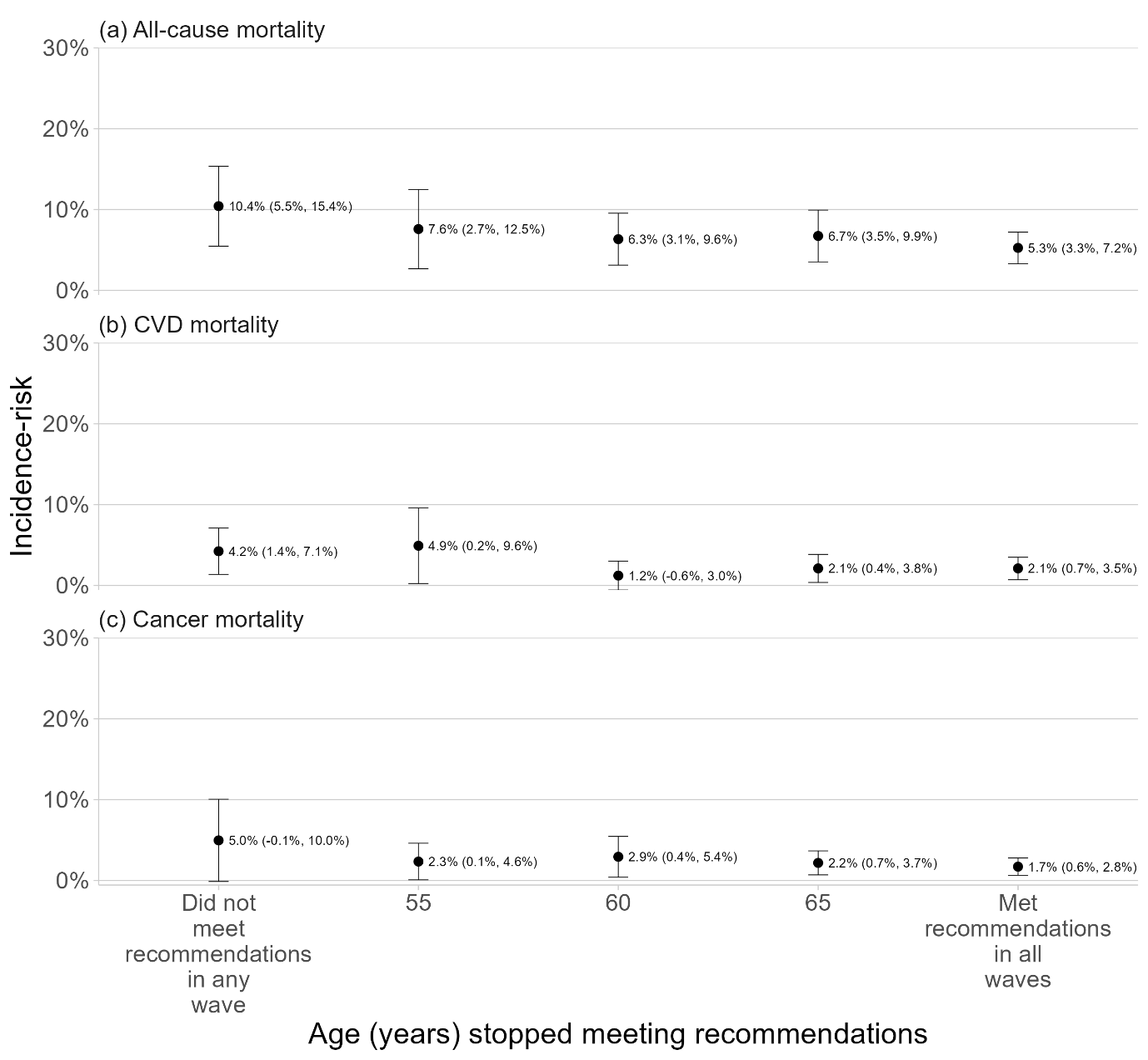


Abbreviations: CVD, cardiovascular disease.

The points represent the estimates and the bars the 99.5% confidence intervals. Numerical values have also been provided for the estimates and the confidence intervals (in parentheses).

Models were adjusted for: highest level of education, country of birth, age, employment status, living with children, marital status, Socio-Economic Index For Areas Index of Relative Socio-Economic Disadvantage (SEIFA IRSD), geographical remoteness (Accessibility-Remoteness Index of Australia Plus, ARIA+), lifetime risky alcohol consumption, heavy episodic alcohol consumption, smoking status, vegetable intake, fruit intake, Center for Epidemiological Studies-Depression (CES-D) scale, perceived stress scale, SF-36 subscale scores, body mass index, diagnosis/treatment history of coronary heart disease, stroke, arthritis, any cancer, anxiety, and depression.

**Fig D in S4 Text** Risk ratio of all-cause, cardiovascular disease (CVD) and cancer mortality linked to different ages of stopping meeting moderate-to-vigorous intensity physical activity (MVPA) recommendations versus not meeting recommendations at all.


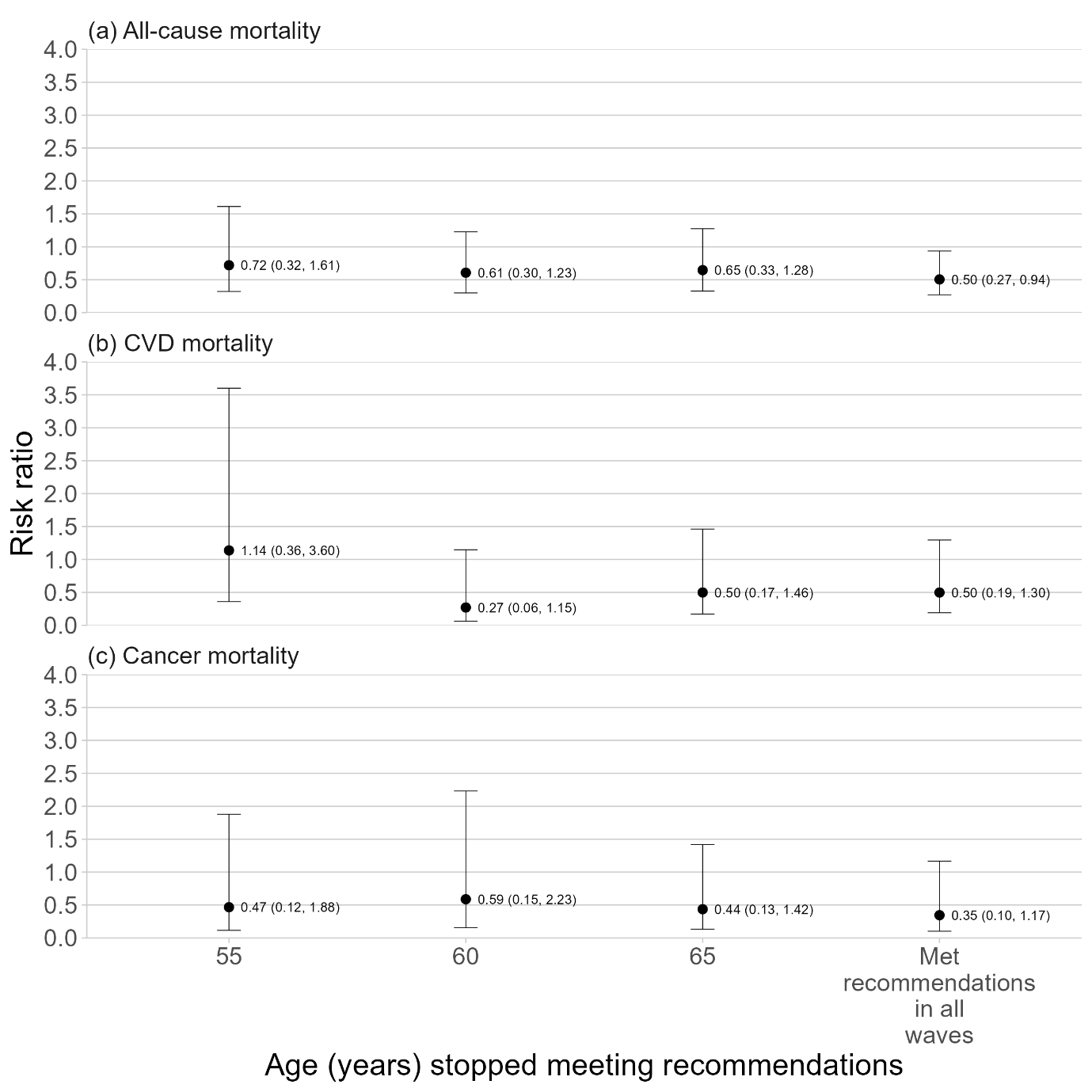


Abbreviations: CVD, cardiovascular disease.

The points represent the estimates and the bars the 99.5% confidence intervals. Numerical values have also been provided for the estimates and the confidence intervals (in parentheses).

Models were adjusted for: highest level of education, country of birth, age, employment status, living with children, marital status, Socio-Economic Index For Areas Index of Relative Socio-Economic Disadvantage (SEIFA IRSD), geographical remoteness (Accessibility-Remoteness Index of Australia Plus, ARIA+), lifetime risky alcohol consumption, heavy episodic alcohol consumption, smoking status, vegetable intake, fruit intake, Center for Epidemiological Studies-Depression (CES-D) scale, perceived stress scale, SF-36 subscale scores, body mass index, diagnosis/treatment history of coronary heart disease, stroke, arthritis, any cancer, anxiety, and depression.

**Fig E in S4 Text** Risk difference of all-cause, cardiovascular disease (CVD) and cancer mortality linked to different ages of stopping meeting moderate-to-vigorous intensity physical activity (MVPA) recommendations versus not meeting recommendations at all.


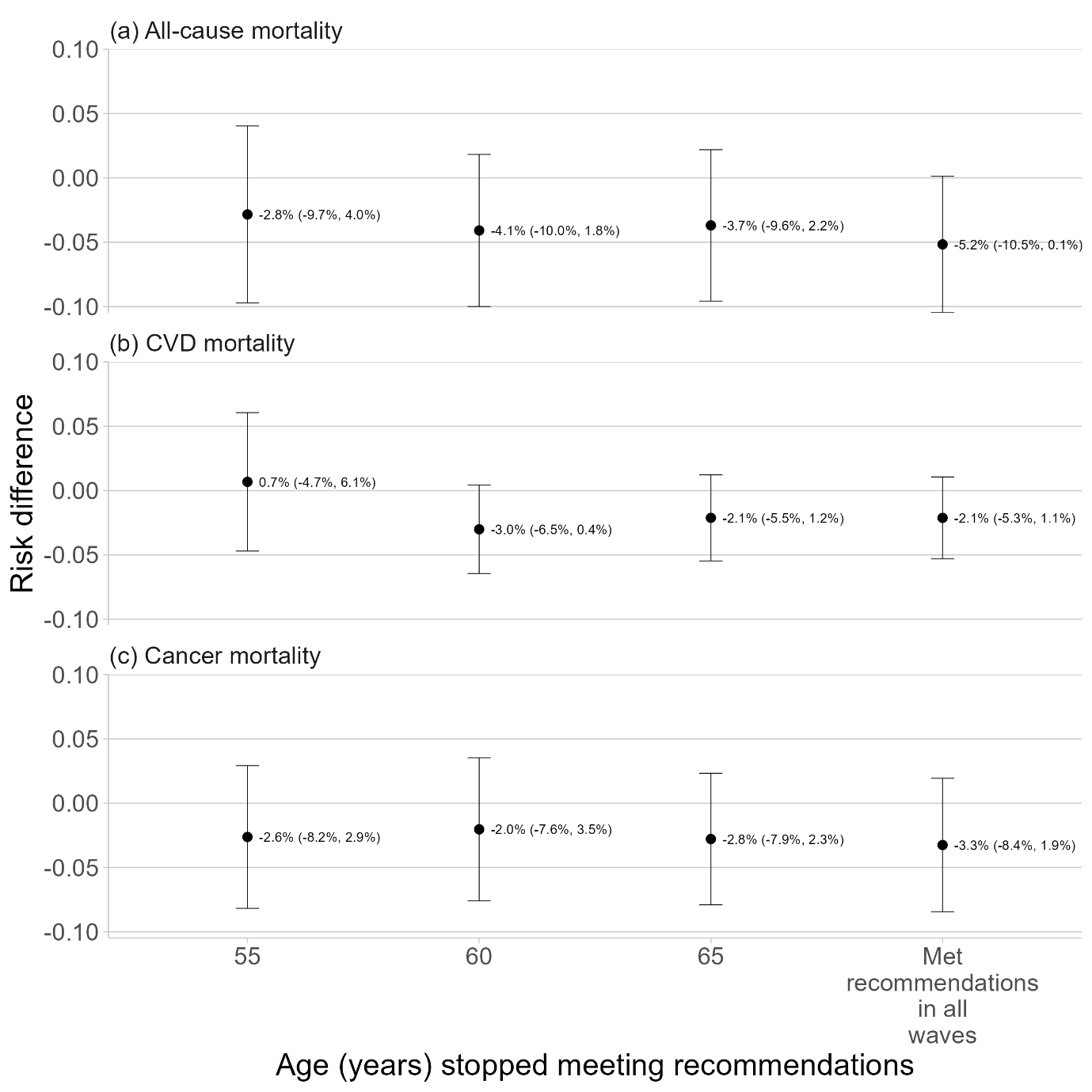


Abbreviations: CVD, cardiovascular disease.

The points represent the estimates and the bars the 99.5% confidence intervals. Numerical values have also been provided for the estimates and the confidence intervals (in parentheses).

Models were adjusted for: highest level of education, country of birth, age, employment status, living with children, marital status, Socio-Economic Index For Areas Index of Relative Socio-Economic Disadvantage (SEIFA IRSD), geographical remoteness (Accessibility-Remoteness Index of Australia Plus, ARIA+), lifetime risky alcohol consumption, heavy episodic alcohol consumption, smoking status, vegetable intake, fruit intake, Center for Epidemiological Studies-Depression (CES-D) scale, perceived stress scale, SF-36 subscale scores, body mass index, diagnosis/treatment history of coronary heart disease, stroke, arthritis, any cancer, anxiety, and depression.

**Fig F in S4 Text** Incidence-risk of all-cause, cardiovascular disease (CVD) and cancer mortality linked to different ages of starting to meet moderate-to-vigorous intensity physical activity (MVPA) recommendations – sensitivity analysis using 75 minutes/day.


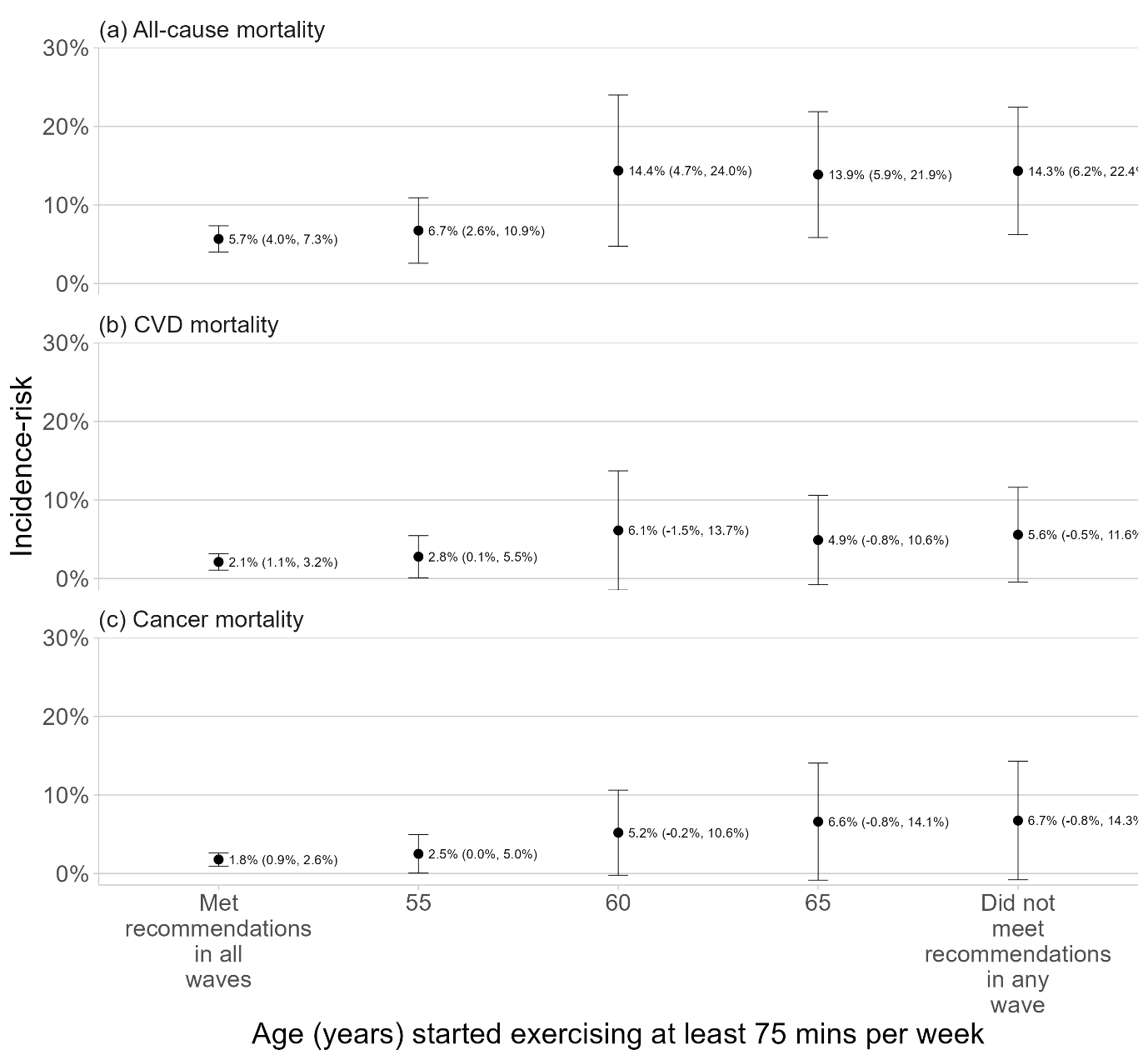


Abbreviations: CVD, cardiovascular disease.

The points represent the estimates and the bars the 99.5% confidence intervals. Numerical values have also been provided for the estimates and the confidence intervals (in parentheses).

Models were adjusted for: highest level of education, country of birth, age, employment status, living with children, marital status, Socio-Economic Index For Areas Index of Relative Socio-Economic Disadvantage (SEIFA IRSD), geographical remoteness (Accessibility-Remoteness Index of Australia Plus, ARIA+), lifetime risky alcohol consumption, heavy episodic alcohol consumption, smoking status, vegetable intake, fruit intake, Center for Epidemiological Studies-Depression (CES-D) scale, perceived stress scale, SF-36 subscale scores, body mass index, diagnosis/treatment history of coronary heart disease, stroke, arthritis, any cancer, anxiety, and depression.

**Fig G in S4 Text** Risk ratio of all-cause, cardiovascular disease (CVD) and cancer mortality linked to different ages of starting to meet moderate-to-vigorous intensity physical activity (MVPA) recommendations versus not meeting recommendations at all – sensitivity analysis using 75 minute/day.


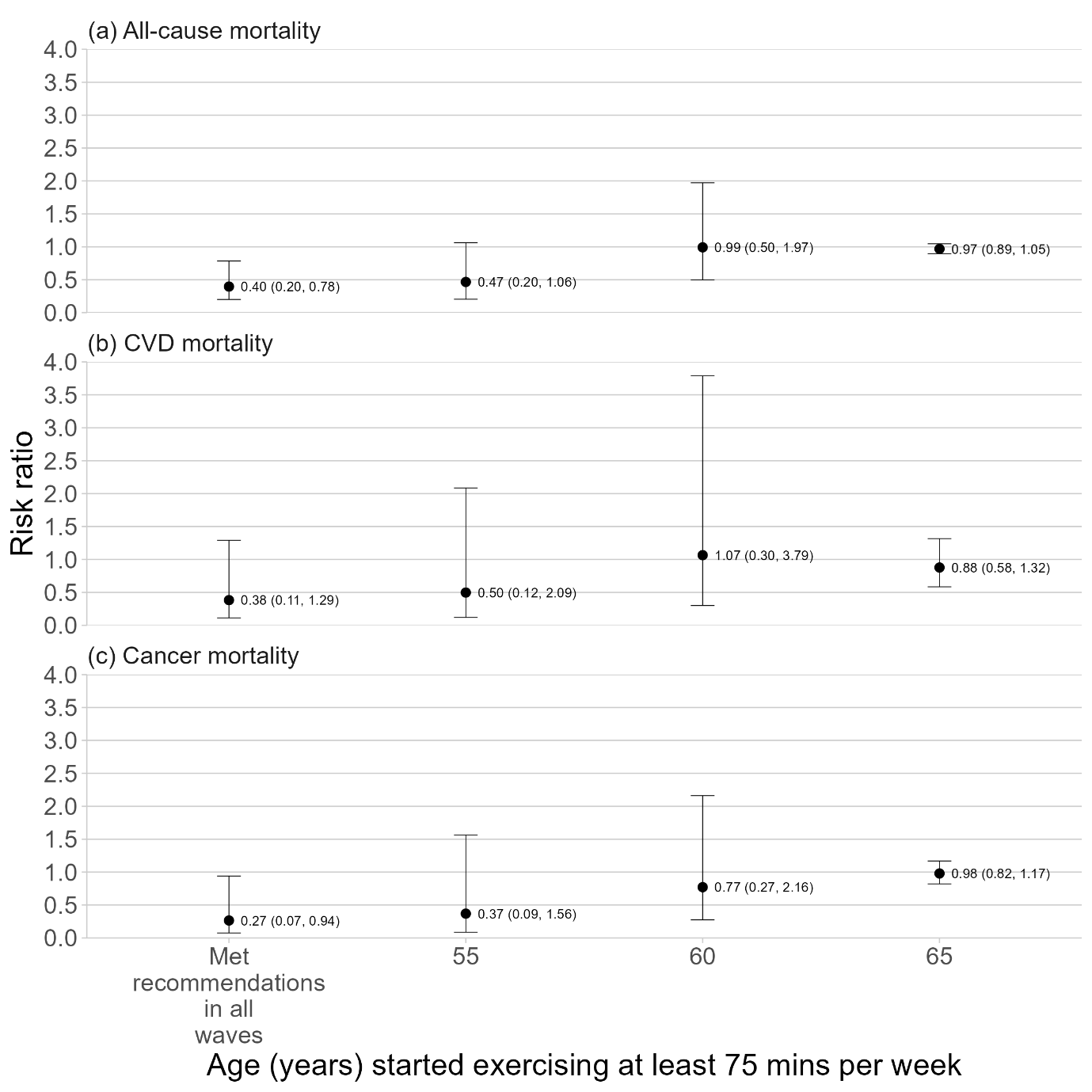


Abbreviations: CVD, cardiovascular disease.

The points represent the estimates and the bars the 99.5% confidence intervals. Numerical values have also been provided for the estimates and the confidence intervals (in parentheses).

Models were adjusted for: highest level of education, country of birth, age, employment status, living with children, marital status, Socio-Economic Index For Areas Index of Relative Socio-Economic Disadvantage (SEIFA IRSD), geographical remoteness (Accessibility-Remoteness Index of Australia Plus, ARIA+), lifetime risky alcohol consumption, heavy episodic alcohol consumption, smoking status, vegetable intake, fruit intake, Center for Epidemiological Studies-Depression (CES-D) scale, perceived stress scale, SF-36 subscale scores, body mass index, diagnosis/treatment history of coronary heart disease, stroke, arthritis, any cancer, anxiety, and depression.

**Fig H in S4 Text** Risk difference of all-cause, cardiovascular disease (CVD) and cancer mortality linked to different ages of starting to meet moderate-to-vigorous intensity physical activity (MVPA) recommendations versus not meeting recommendations at all – sensitivity analysis using 75 minute/day.


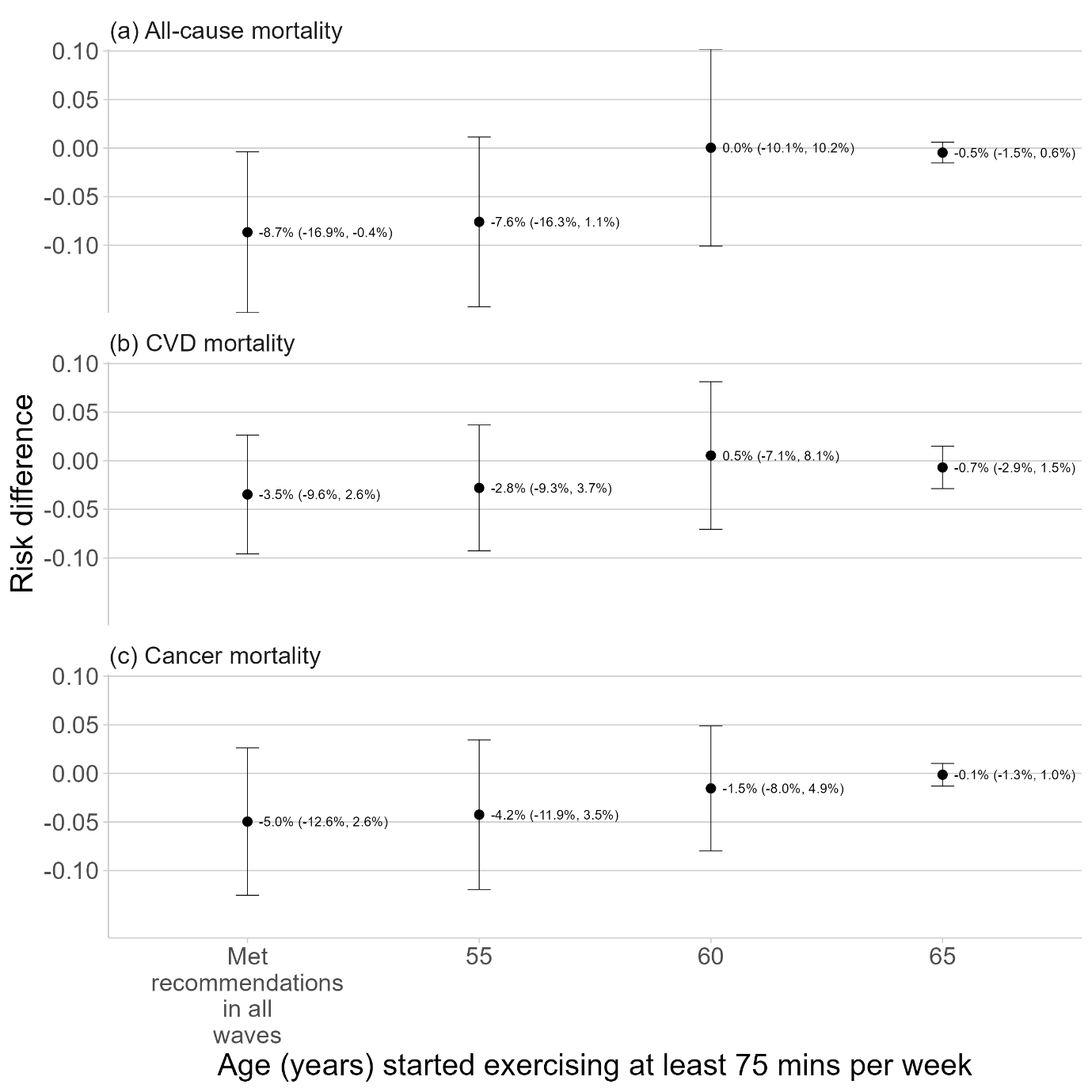


Abbreviations: CVD, cardiovascular disease.

The points represent the estimates and the bars the 99.5% confidence intervals. Numerical values have also been provided for the estimates and the confidence intervals (in parentheses).

Models were adjusted for: highest level of education, country of birth, age, employment status, living with children, marital status, Socio-Economic Index For Areas Index of Relative Socio-Economic Disadvantage (SEIFA IRSD), geographical remoteness (Accessibility-Remoteness Index of Australia Plus, ARIA+), lifetime risky alcohol consumption, heavy episodic alcohol consumption, smoking status, vegetable intake, fruit intake, Center for Epidemiological Studies-Depression (CES-D) scale, perceived stress scale, SF-36 subscale scores, body mass index, diagnosis/treatment history of coronary heart disease, stroke, arthritis, any cancer, anxiety, and depression.

**Fig I in S4 Text** Incidence-risk of all-cause, cardiovascular disease (CVD) and cancer mortality linked to different ages of stopping meeting moderate-to-vigorous intensity physical activity (MVPA) recommendations – sensitivity analysis using 75 minutes/day.


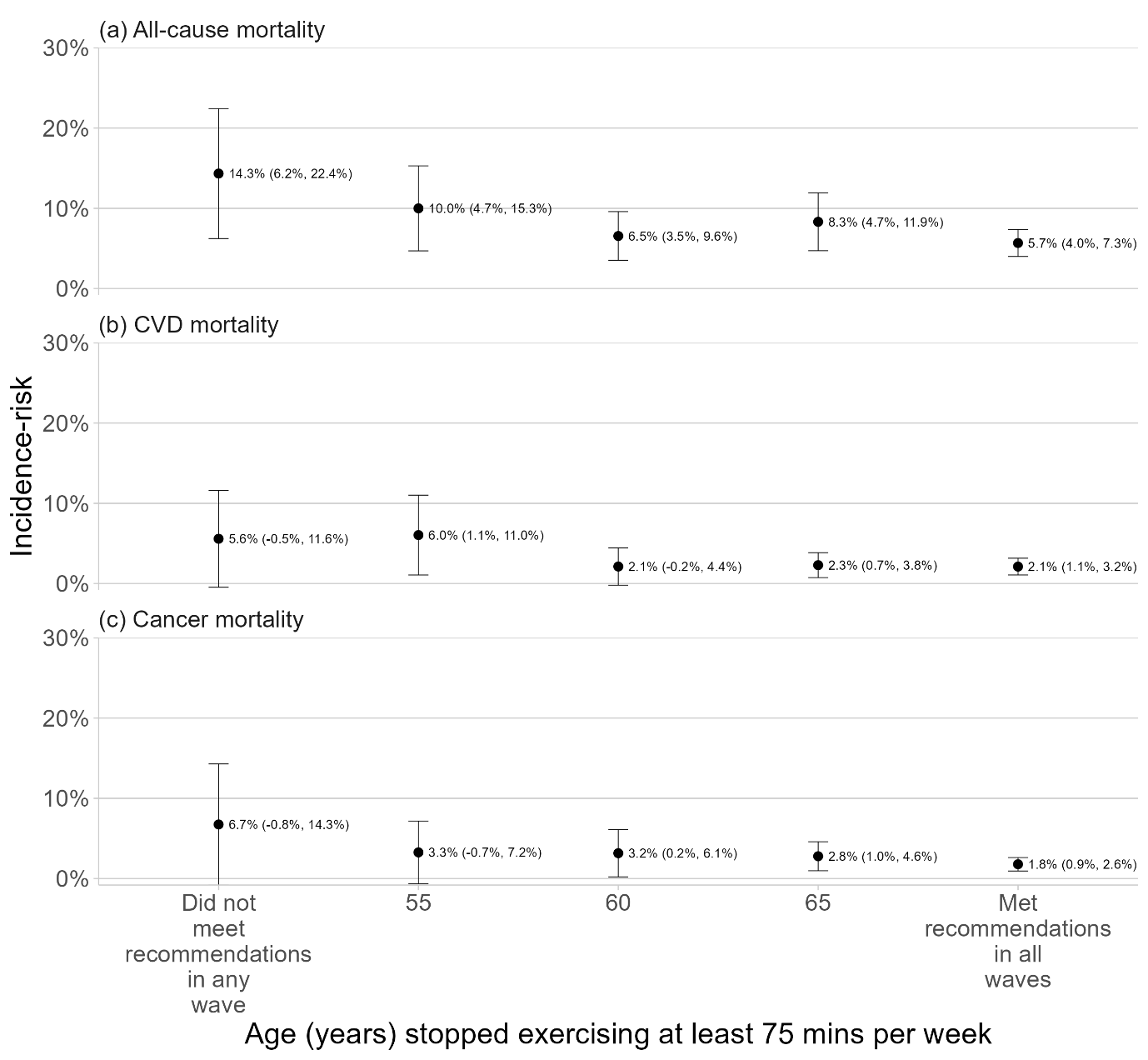


Abbreviations: CVD, cardiovascular disease.

The points represent the estimates and the bars the 99.5% confidence intervals. Numerical values have also been provided for the estimates and the confidence intervals (in parentheses).

Models were adjusted for: highest level of education, country of birth, age, employment status, living with children, marital status, Socio-Economic Index For Areas Index of Relative Socio-Economic Disadvantage (SEIFA IRSD), geographical remoteness (Accessibility-Remoteness Index of Australia Plus, ARIA+), lifetime risky alcohol consumption, heavy episodic alcohol consumption, smoking status, vegetable intake, fruit intake, Center for Epidemiological Studies-Depression (CES-D) scale, perceived stress scale, SF-36 subscale scores, body mass index, diagnosis/treatment history of coronary heart disease, stroke, arthritis, any cancer, anxiety, and depression.

**Fig J in S4 Text** Risk ratio of all-cause, cardiovascular disease (CVD) and cancer mortality linked to different ages of stopping meeting moderate-to-vigorous intensity physical activity (MVPA) recommendations versus not meeting recommendations at all – sensitivity analysis using 75 minutes/day.


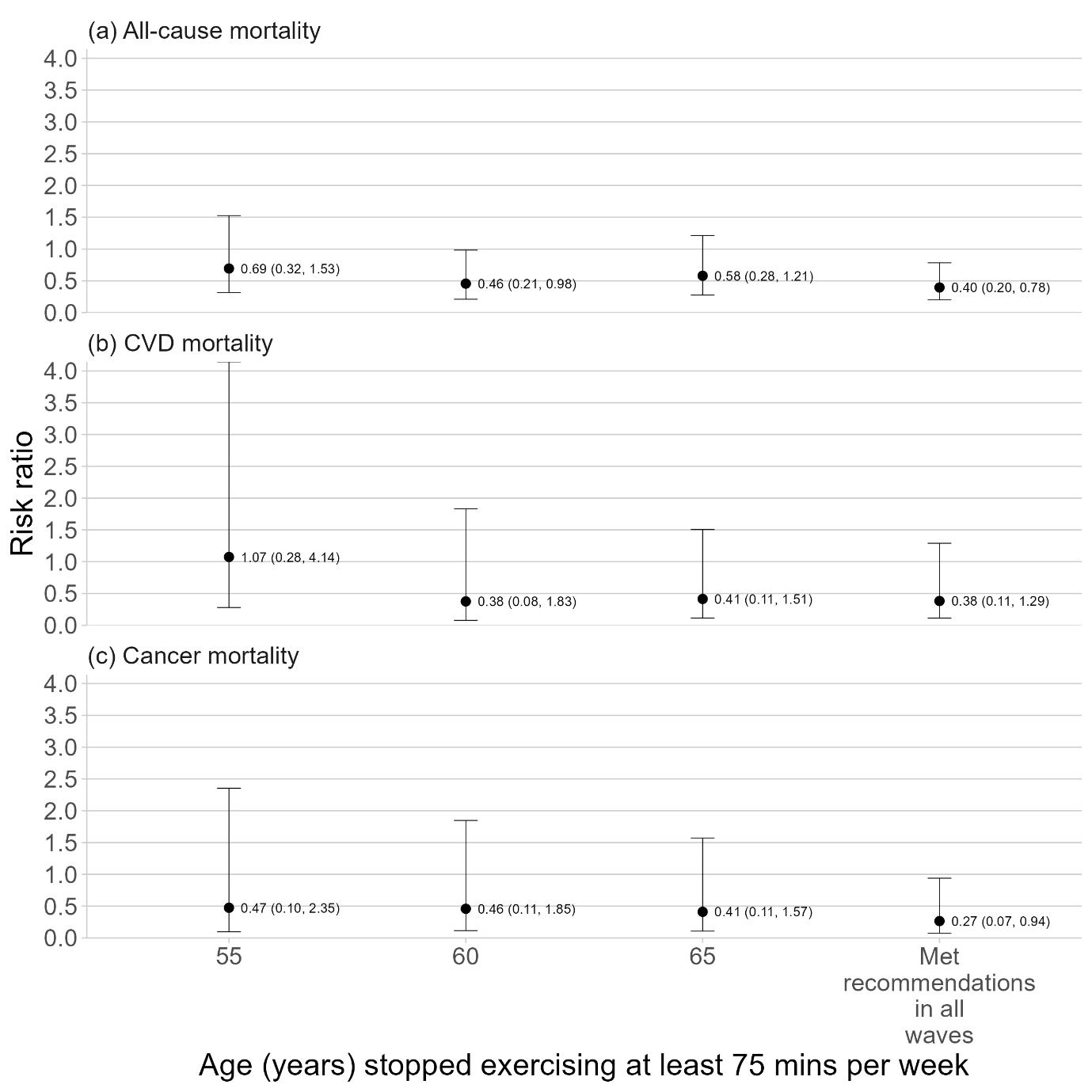


Abbreviations: CVD, cardiovascular disease.

The points represent the estimates and the bars the 99.5% confidence intervals. Numerical values have also been provided for the estimates and the confidence intervals (in parentheses).

Models were adjusted for: highest level of education, country of birth, age, employment status, living with children, marital status, Socio-Economic Index For Areas Index of Relative Socio-Economic Disadvantage (SEIFA IRSD), geographical remoteness (Accessibility-Remoteness Index of Australia Plus, ARIA+), lifetime risky alcohol consumption, heavy episodic alcohol consumption, smoking status, vegetable intake, fruit intake, Center for Epidemiological Studies-Depression (CES-D) scale, perceived stress scale, SF-36 subscale scores, body mass index, diagnosis/treatment history of coronary heart disease, stroke, arthritis, any cancer, anxiety, and depression.

**Fig K in S4 Text** Risk difference of all-cause, cardiovascular disease (CVD) and cancer mortality linked to different ages of stopping meeting moderate-to-vigorous intensity physical activity (MVPA) recommendations versus not meeting recommendations at all – sensitivity analysis using 75 minutes/day.


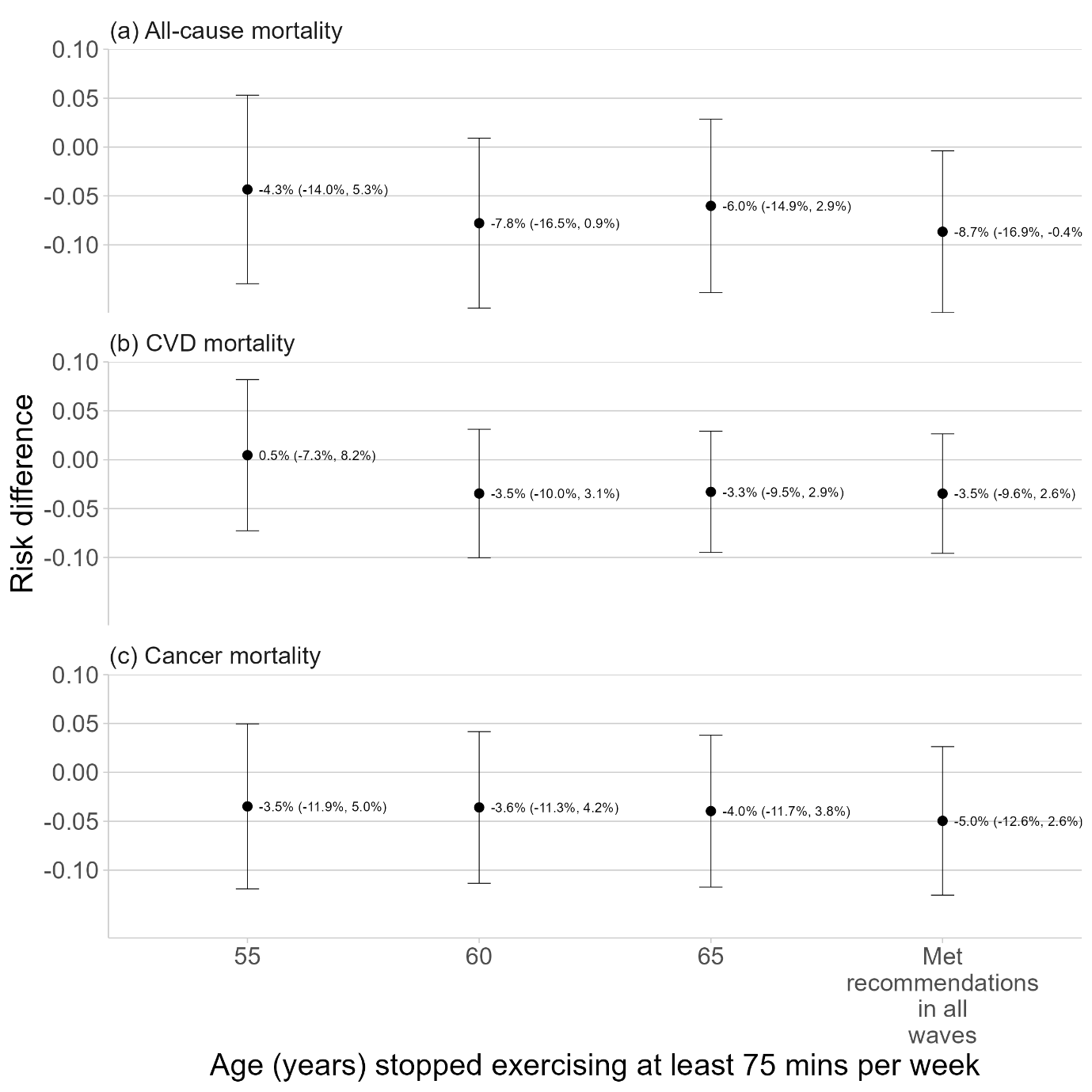


Abbreviations: CVD, cardiovascular disease.

The points represent the estimates and the bars the 99.5% confidence intervals. Numerical values have also been provided for the estimates and the confidence intervals (in parentheses).

Models were adjusted for: highest level of education, country of birth, age, employment status, living with children, marital status, Socio-Economic Index For Areas Index of Relative Socio-Economic Disadvantage (SEIFA IRSD), geographical remoteness (Accessibility-Remoteness Index of Australia Plus, ARIA+), lifetime risky alcohol consumption, heavy episodic alcohol consumption, smoking status, vegetable intake, fruit intake, Center for Epidemiological Studies-Depression (CES-D) scale, perceived stress scale, SF-36 subscale scores, body mass index, diagnosis/treatment history of coronary heart disease, stroke, arthritis, any cancer, anxiety, and depression.

**Fig L in S4 Text** Incidence-risk of all-cause, cardiovascular disease (CVD) and cancer mortality linked to different ages of starting to meet moderate-to-vigorous intensity physical activity (MVPA) recommendations – sensitivity analysis using 300 minutes/day.


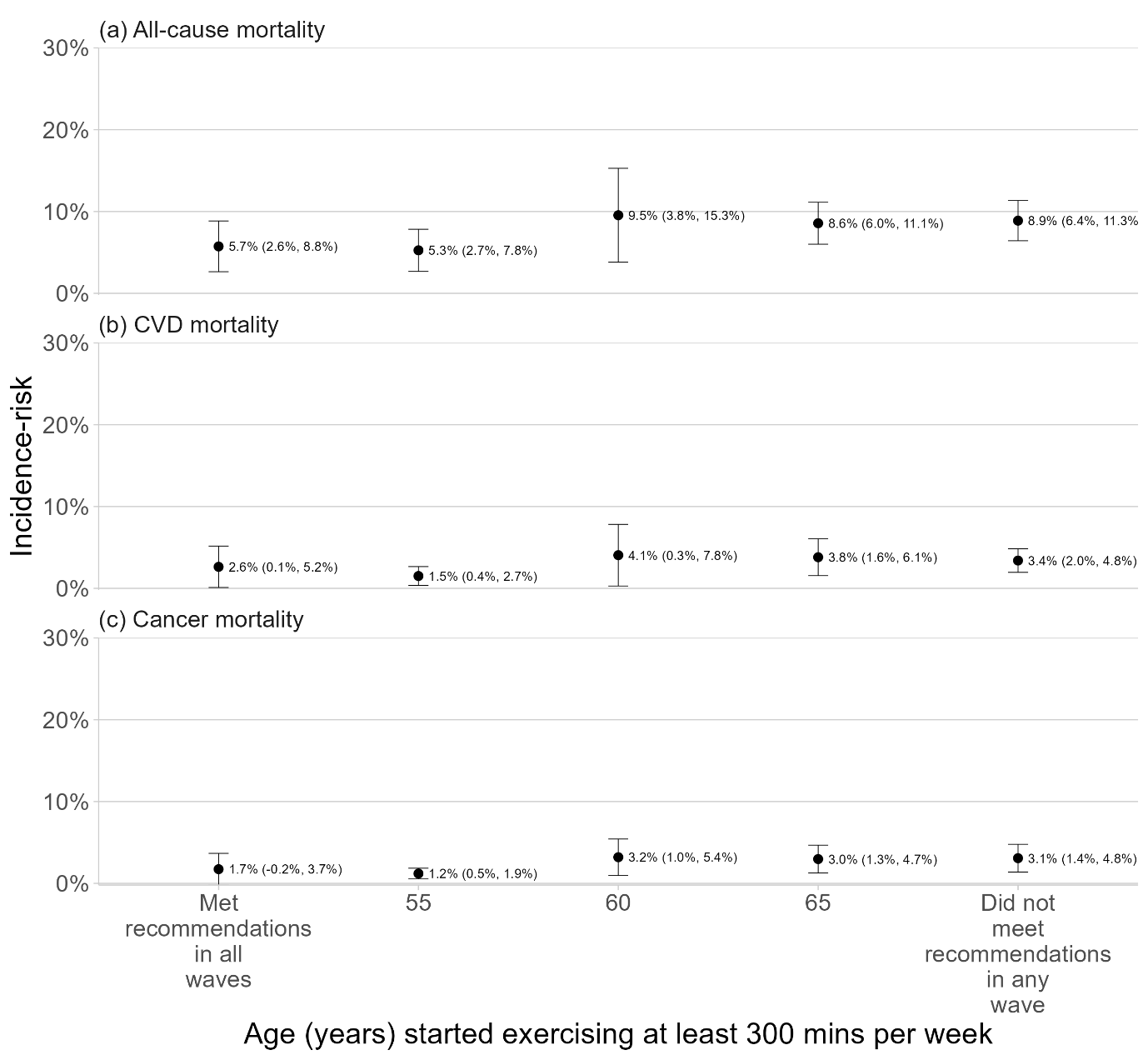


Abbreviations: CVD, cardiovascular disease.

The points represent the estimates and the bars the 99.5% confidence intervals. Numerical values have also been provided for the estimates and the confidence intervals (in parentheses).

Models were adjusted for: highest level of education, country of birth, age, employment status, living with children, marital status, Socio-Economic Index For Areas Index of Relative Socio-Economic Disadvantage (SEIFA IRSD), geographical remoteness (Accessibility-Remoteness Index of Australia Plus, ARIA+), lifetime risky alcohol consumption, heavy episodic alcohol consumption, smoking status, vegetable intake, fruit intake, Center for Epidemiological Studies-Depression (CES-D) scale, perceived stress scale, SF-36 subscale scores, body mass index, diagnosis/treatment history of coronary heart disease, stroke, arthritis, any cancer, anxiety, and depression.

**Fig M in S4 Text** Risk ratio of all-cause, cardiovascular disease (CVD) and cancer mortality linked to different ages of starting to meet moderate-to-vigorous intensity physical activity (MVPA) recommendations versus not meeting recommendations at all – sensitivity analysis using 300 minutes/day.


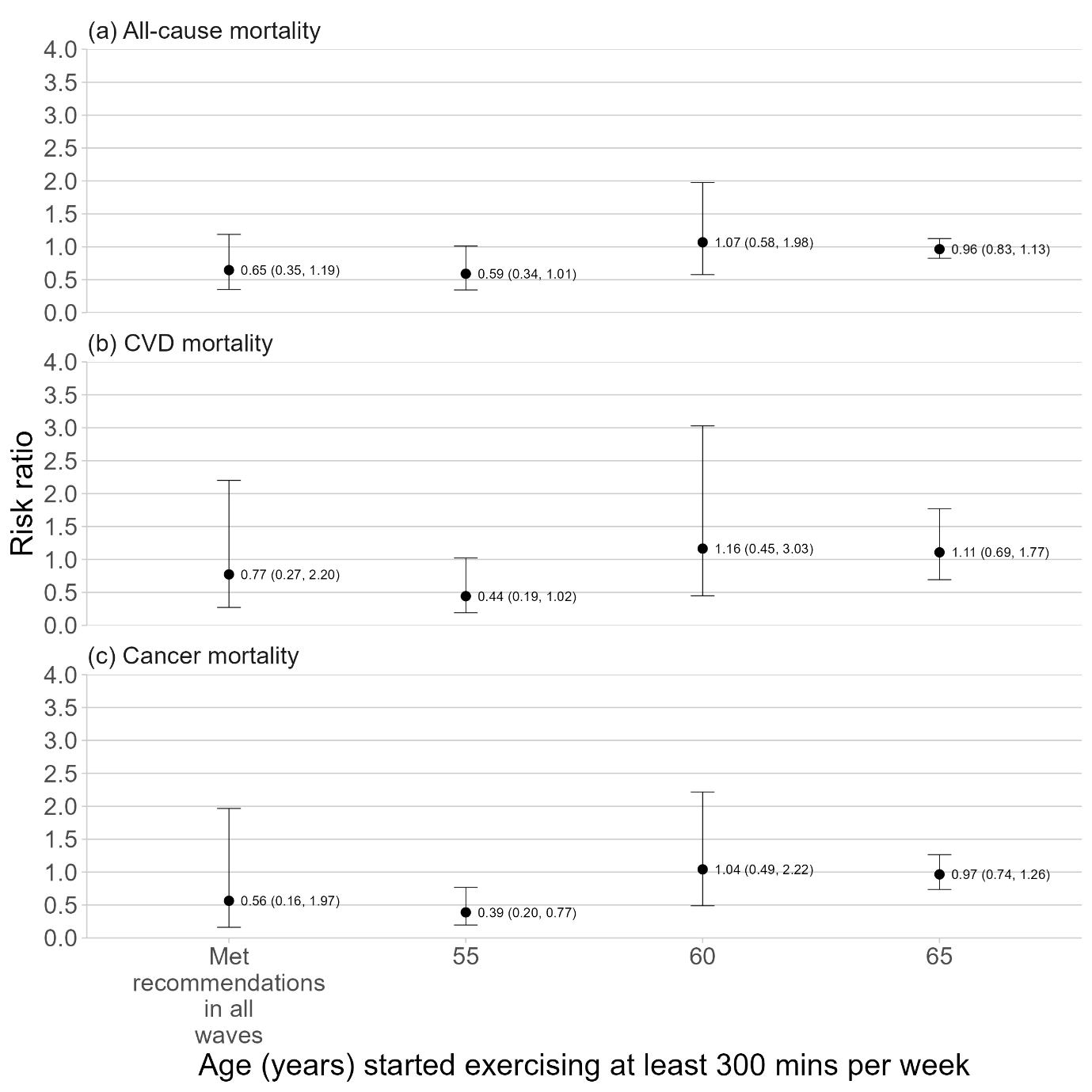


Abbreviations: CVD, cardiovascular disease.

The points represent the estimates and the bars the 99.5% confidence intervals. Numerical values have also been provided for the estimates and the confidence intervals (in parentheses).

Models were adjusted for: highest level of education, country of birth, age, employment status, living with children, marital status, Socio-Economic Index For Areas Index of Relative Socio-Economic Disadvantage (SEIFA IRSD), geographical remoteness (Accessibility-Remoteness Index of Australia Plus, ARIA+), lifetime risky alcohol consumption, heavy episodic alcohol consumption, smoking status, vegetable intake, fruit intake, Center for Epidemiological Studies-Depression (CES-D) scale, perceived stress scale, SF-36 subscale scores, body mass index, diagnosis/treatment history of coronary heart disease, stroke, arthritis, any cancer, anxiety, and depression.

**Fig N in S4 Text** Risk difference of all-cause, cardiovascular disease (CVD) and cancer mortality linked to different ages of starting to meet moderate-to-vigorous intensity physical activity (MVPA) recommendations versus not meeting recommendations at all – sensitivity analysis using 300 minutes/day.


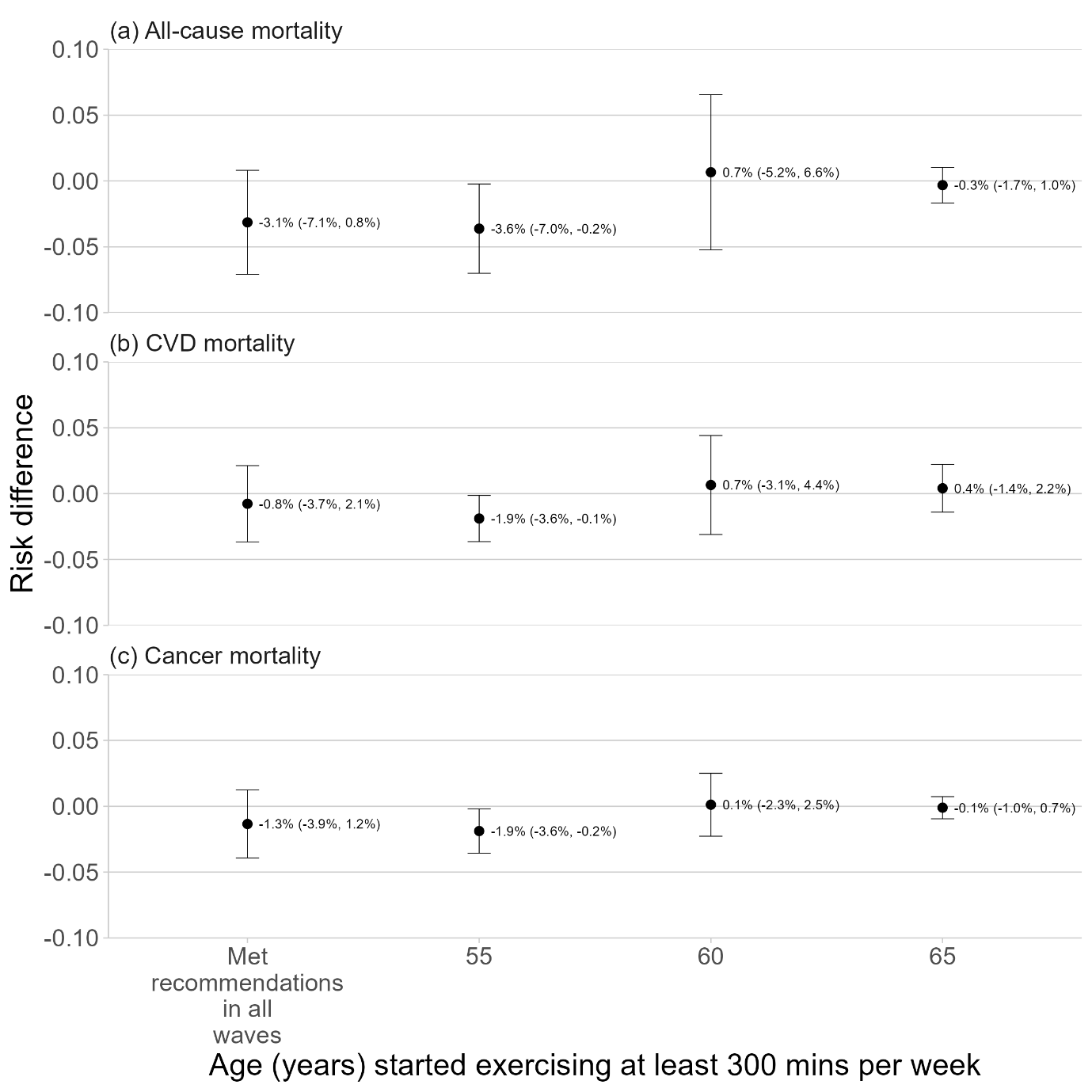


Abbreviations: CVD, cardiovascular disease.

The points represent the estimates and the bars the 99.5% confidence intervals. Numerical values have also been provided for the estimates and the confidence intervals (in parentheses).

Models were adjusted for: highest level of education, country of birth, age, employment status, living with children, marital status, Socio-Economic Index For Areas Index of Relative Socio-Economic Disadvantage (SEIFA IRSD), geographical remoteness (Accessibility-Remoteness Index of Australia Plus, ARIA+), lifetime risky alcohol consumption, heavy episodic alcohol consumption, smoking status, vegetable intake, fruit intake, Center for Epidemiological Studies-Depression (CES-D) scale, perceived stress scale, SF-36 subscale scores, body mass index, diagnosis/treatment history of coronary heart disease, stroke, arthritis, any cancer, anxiety, and depression.

**Fig O in S4 Text** Incidence-risk of all-cause, cardiovascular disease (CVD) and cancer mortality linked to different ages of stopping meeting moderate-to-vigorous intensity physical activity (MVPA) recommendations – sensitivity analysis using 300 minutes/day.


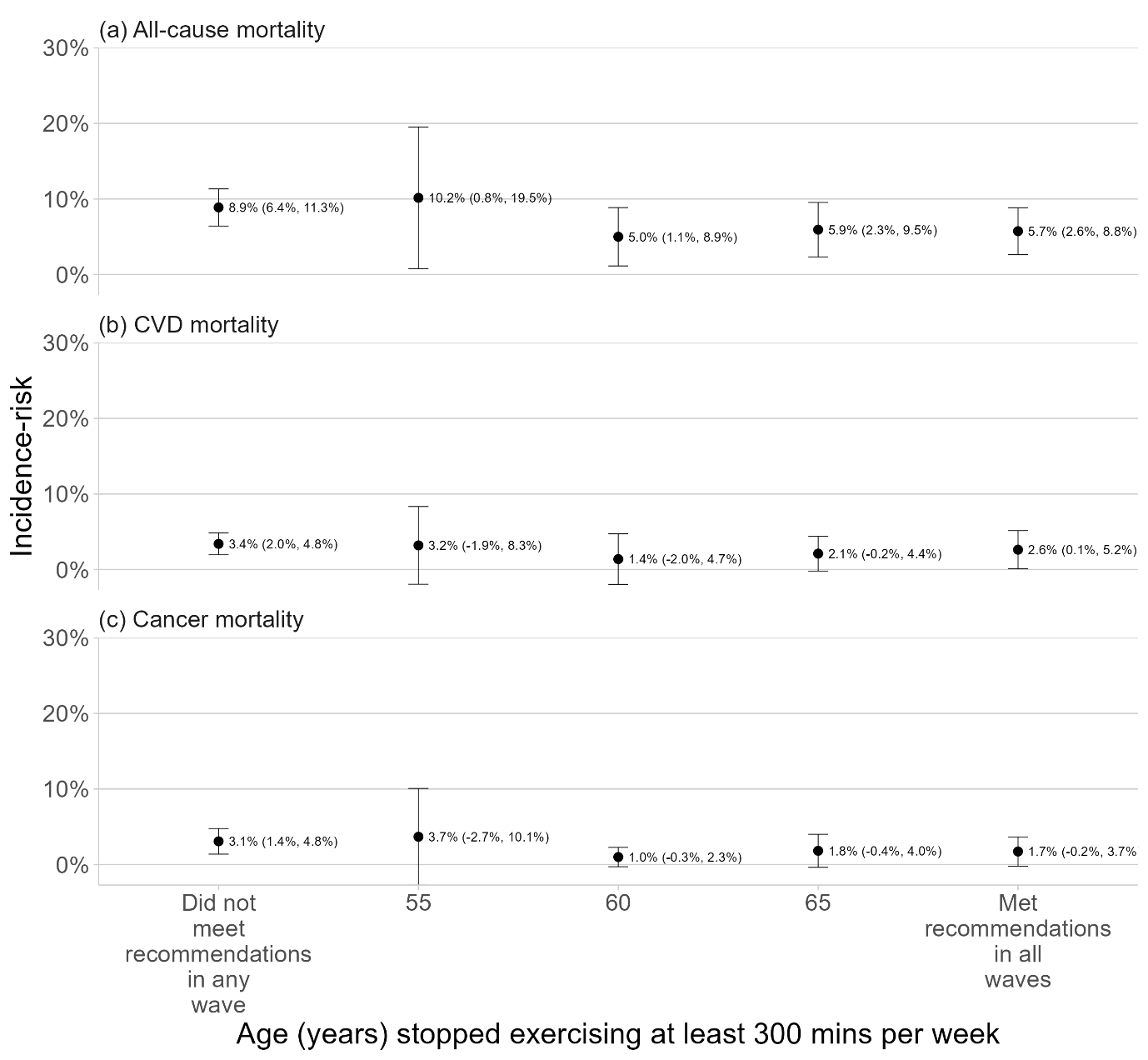


Abbreviations: CVD, cardiovascular disease.

The points represent the estimates and the bars the 99.5% confidence intervals. Numerical values have also been provided for the estimates and the confidence intervals (in parentheses).

Models were adjusted for: highest level of education, country of birth, age, employment status, living with children, marital status, Socio-Economic Index For Areas Index of Relative Socio-Economic Disadvantage (SEIFA IRSD), geographical remoteness (Accessibility-Remoteness Index of Australia Plus, ARIA+), lifetime risky alcohol consumption, heavy episodic alcohol consumption, smoking status, vegetable intake, fruit intake, Center for Epidemiological Studies-Depression (CES-D) scale, perceived stress scale, SF-36 subscale scores, body mass index, diagnosis/treatment history of coronary heart disease, stroke, arthritis, any cancer, anxiety, and depression.

**Fig P in S4 Text** Risk ratio of all-cause, cardiovascular disease (CVD) and cancer mortality linked to different ages of stopping to meet moderate-to-vigorous intensity physical activity (MVPA) recommendations versus not meeting recommendations at all – sensitivity analysis using 300 minutes/day.


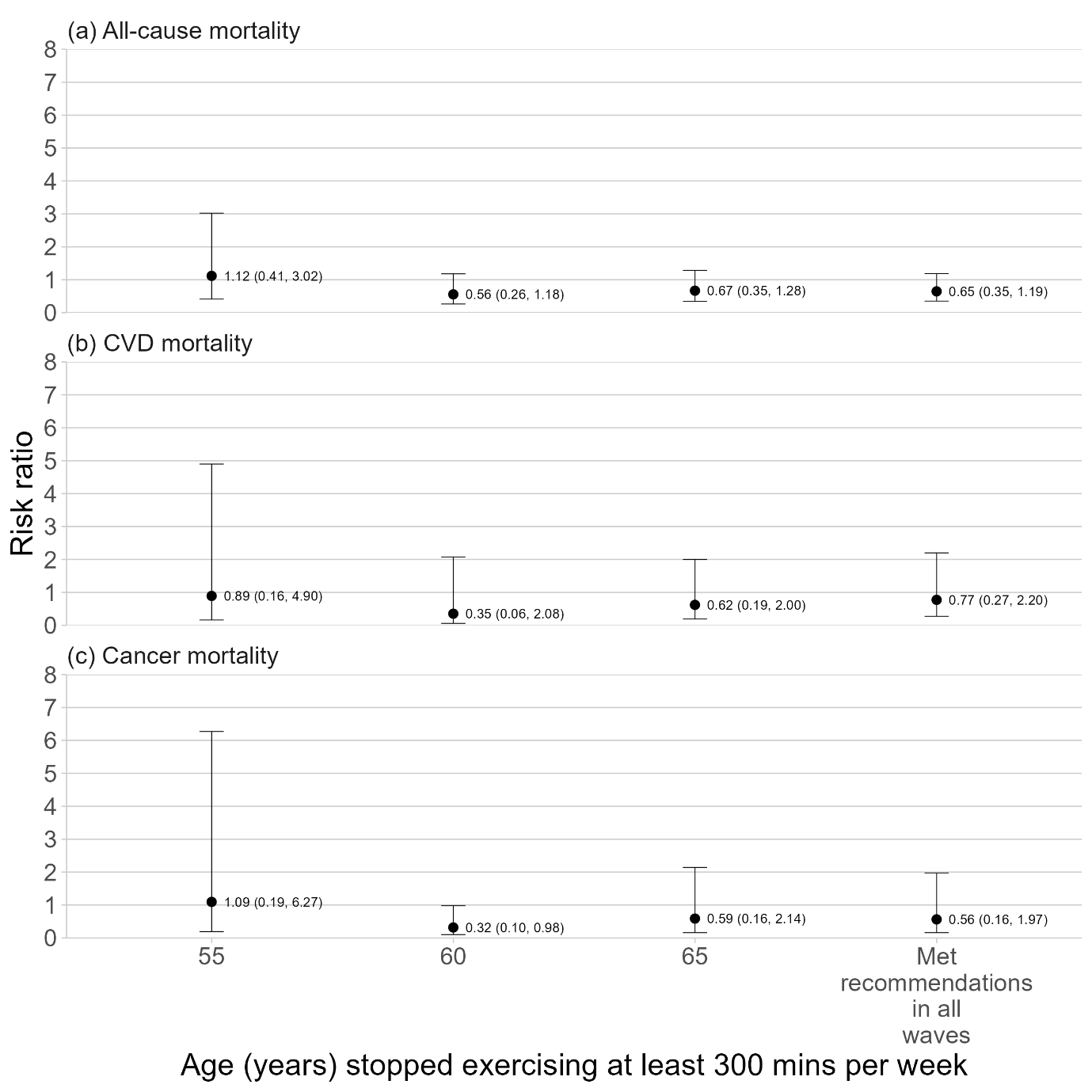


Abbreviations: CVD, cardiovascular disease.

The points represent the estimates and the bars the 99.5% confidence intervals. Numerical values have also been provided for the estimates and the confidence intervals (in parentheses).

Models were adjusted for: highest level of education, country of birth, age, employment status, living with children, marital status, Socio-Economic Index For Areas Index of Relative Socio-Economic Disadvantage (SEIFA IRSD), geographical remoteness (Accessibility-Remoteness Index of Australia Plus, ARIA+), lifetime risky alcohol consumption, heavy episodic alcohol consumption, smoking status, vegetable intake, fruit intake, Center for Epidemiological Studies-Depression (CES-D) scale, perceived stress scale, SF-36 subscale scores, body mass index, diagnosis/treatment history of coronary heart disease, stroke, arthritis, any cancer, anxiety, and depression.

**Fig Q in S4 Text** Risk difference of all-cause, cardiovascular disease (CVD) and cancer mortality linked to different ages of stopping to meet moderate-to-vigorous intensity physical activity (MVPA) recommendations versus not meeting recommendations at all – sensitivity analysis using 300 minutes/day.


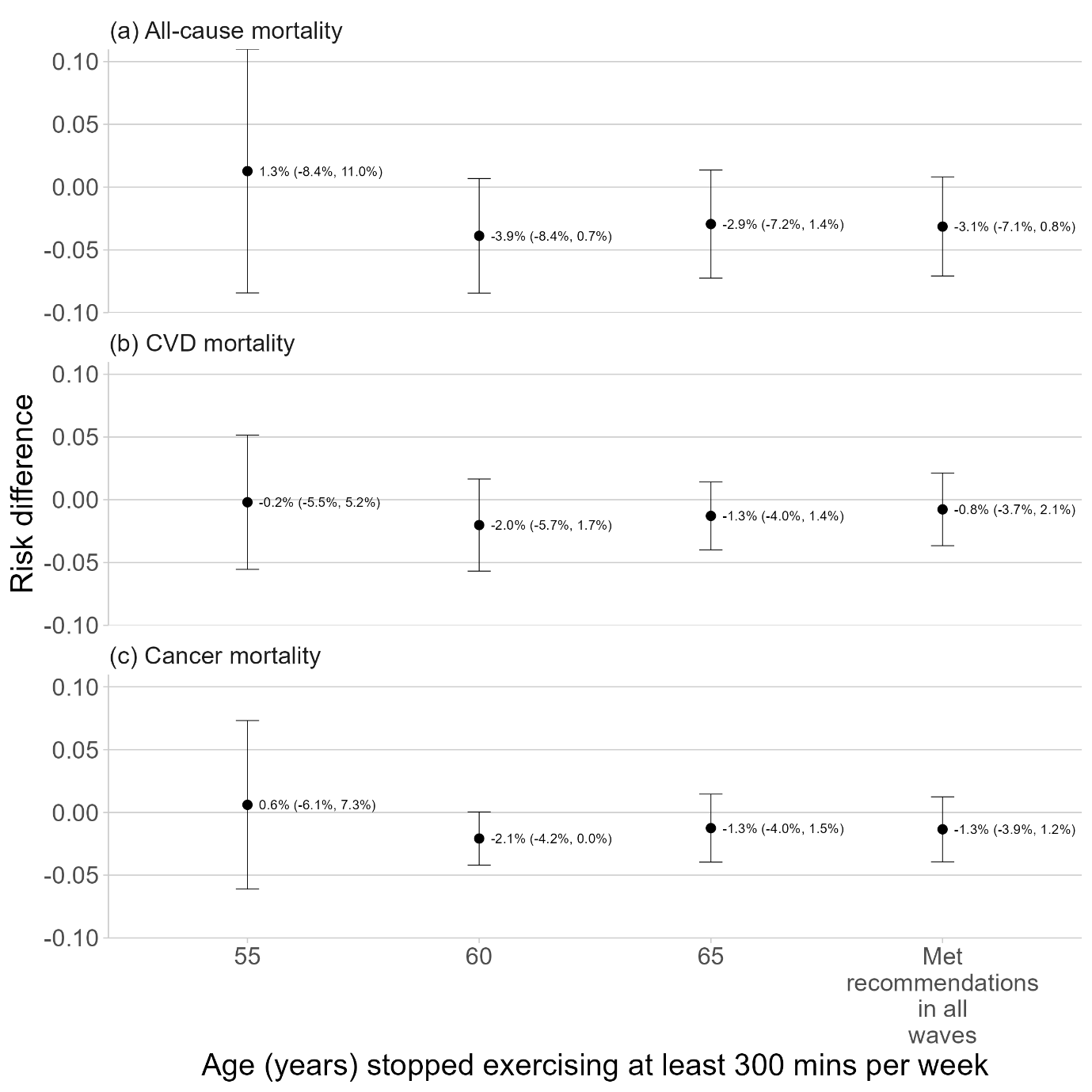


Abbreviations: CVD, cardiovascular disease.

The points represent the estimates and the bars the 99.5% confidence intervals. Numerical values have also been provided for the estimates and the confidence intervals (in parentheses).

Models were adjusted for: highest level of education, country of birth, age, employment status, living with children, marital status, Socio-Economic Index For Areas Index of Relative Socio-Economic Disadvantage (SEIFA IRSD), geographical remoteness (Accessibility-Remoteness Index of Australia Plus, ARIA+), lifetime risky alcohol consumption, heavy episodic alcohol consumption, smoking status, vegetable intake, fruit intake, Center for Epidemiological Studies-Depression (CES-D) scale, perceived stress scale, SF-36 subscale scores, body mass index, diagnosis/treatment history of coronary heart disease, stroke, arthritis, any cancer, anxiety, and depression.

**Table B in S4 Text** E-Value analysis for primary and secondary analyses.

| **Counterfactual** | | **All-cause mortality** | | **CVD mortality** | | **Cancer mortality** | |  |
| --- | --- | --- | --- | --- | --- | --- | --- | --- |
|  |  | **At estimate** | **At inner CI bound** | **At estimate** | **At inner CI bound** | **At estimate** | **At inner CI bound** |  |
| Age started meeting recommendations | Met recommendations in all waves | 3.38 | 1.34 | 3.43 | 1.00 | 5.24 | 1.00 |  |
|  | 55 | 2.62 | 1.00 | 2.99 | 1.00 | 3.32 | 1.00 |  |
|  | 60 | 1.87 | 1.00 | 2.10 | 1.00 | 1.17 | 1.00 |  |
|  | 65 | 1.33 | 1.00 | 1.45 | 1.00 | 1.14 | 1.00 |  |
|  | Did not meet recommendations in any wave | REF | REF | REF | REF | REF | REF |  |
| Age stopped meeting recommendations | | Did not meet recommendations in any wave | REF | REF | REF | REF | REF | REF |
|  |  | 55 | 2.12 | 1.00 | 2.12 | 1.00 | 3.71 | 1.00 |
|  |  | 60 | 2.68 | 1.00 | 2.68 | 1.00 | 2.79 | 1.00 |
|  |  | 65 | 2.47 | 1.00 | 2.47 | 1.00 | 4.01 | 1.00 |
|  |  | Met recommendations in all waves | 3.38 | 1.34 | 3.38 | 1.34 | 5.24 | 1.00 |

Abbreviations: CI, confidence interval; CVD, cardiovascular disease.

Note: E-Value is 1.0 when CI crosses null as no additional confounding is needed to result in inconclusive findings.

**Table C in S4 Text** E-Value analysis for sensitivity analysis using 75 minutes/day.

| **Counterfactual** | | **All-cause mortality** | | **CVD mortality** | | **Cancer mortality** | |
| --- | --- | --- | --- | --- | --- | --- | --- |
|  |  | **At estimate** | **At inner CI bound** | **At estimate** | **At inner CI bound** | **At estimate** | **At inner CI bound** |
| Age started meeting recommendations | Met recommendations in all waves | 4.49 | 1.87 | 4.67 | 1.00 | 7.01 | 1.33 |
|  | 55 | 3.71 | 1.00 | 3.44 | 1.00 | 4.86 | 1.00 |
|  | 60 | 1.10 | 1.00 | 1.33 | 1.00 | 1.92 | 1.00 |
|  | 65 | 1.22 | 1.00 | 1.54 | 1.00 | 1.17 | 1.00 |
|  | Did not meet recommendations in any wave | REF | REF | REF | REF | REF | REF |
| Age stopped meeting recommendations | Did not meet recommendations in any wave | REF | REF | REF | REF | REF | REF |
|  | 55 | 2.24 | 1.00 | 1.36 | 1.00 | 3.63 | 1.00 |
|  | 60 | 3.81 | 1.14 | 4.77 | 1.00 | 3.78 | 1.00 |
|  | 65 | 2.84 | 1.00 | 4.27 | 1.00 | 4.30 | 1.00 |
|  | Met recommendations in all waves | 4.49 | 1.87 | 4.67 | 1.00 | 7.01 | 1.33 |

Abbreviations: CI, confidence interval; CVD, cardiovascular disease.

Note: E-Value is 1.0 when CI crosses null as no additional confounding is needed to result in inconclusive findings.

**Table D in S4 Text** E-Value analysis for sensitivity analysis using 300 minutes/day.

| **Counterfactual** | | **All-cause mortality** | | **CVD mortality** | | **Cancer mortality** | |
| --- | --- | --- | --- | --- | --- | --- | --- |
|  |  | **At estimate** | **At inner CI bound** | **At estimate** | **At inner CI bound** | **At estimate** | **At inner CI bound** |
| Age started meeting recommendations | Met recommendations in all waves | 1.20 | 1.06 | 1.09 | 1.00 | 1.20 | 1.06 |
|  | 55 | 1.22 | 1.12 | 1.15 | 1.08 | 1.22 | 1.12 |
|  | 60 | 1.08 | 1.00 | 1.08 | 1.00 | 1.08 | 1.00 |
|  | 65 | 1.06 | 1.00 | 1.06 | 1.00 | 1.06 | 1.00 |
|  | Did not meet recommendations in any wave | REF | REF | REF | REF | REF | REF |
| Age stopped  meeting recommendations | Did not meet recommendations in any wave | REF | REF | REF | REF | REF | REF |
|  | 55 | 1.48 | 1.00 | 1.48 | 1.00 | 1.42 | 1.00 |
|  | 60 | 3.00 | 1.00 | 3.00 | 1.00 | 5.75 | 1.16 |
|  | 65 | 2.36 | 1.00 | 2.36 | 1.00 | 2.79 | 1.00 |
|  | Met recommendations in all waves | 2.47 | 1.00 | 2.47 | 1.00 | 2.94 | 1.00 |

Abbreviations: CI, confidence interval; CVD, cardiovascular disease.

Note: E-Value is 1.0 when CI crosses null as no additional confounding is needed to result in inconclusive findings.
